# A two-stage hierarchical support vector machine framework detects roasted coffee adulterants through principal component analysis of hyperspectral imaging data

**DOI:** 10.1038/s41598-026-60975-z

**Published:** 2026-07-09

**Authors:** Ahmed AL-Agouz, Mohamed Ebrahem, Mohamed I. Hosni, Adel Abdallah, Alaaeldin Mahmoud

**Affiliations:** 1 Optoelectronics and Automatic Control Systems Department, Military Technical College, Cairo, Egypt; 2 Naval Warning Engineering Department, Military Technical College, Cairo, Egypt

**Keywords:** Hyperspectral imaging, Support vector machine, Principal component analysis, Forensic food authentication, Maillard reaction, Food safety, Chemistry, Engineering, Mathematics and computing

## Abstract

Economically motivated adulteration (EMA) of roasted coffee presents a critical challenge to global market integrity and consumer safety, specifically regarding the surreptitious inclusion of high-risk allergens like barley and soybeans. The detection of such contaminants is historically hindered by the Maillard reaction, a thermal convergence during roasting that renders adulterants visually and spectrally indistinguishable from the coffee matrix. To address this forensic gap, this study presents a targeted hyperspectral imaging (HSI) framework (400–1000 nm) integrated with a two-stage hierarchical Support Vector Machine (SVM) designed to decouple detection from specific biological diagnosis. A core contribution of this methodology is the implementation of Principal Component Analysis (PCA) to resolve spectral redundancy across the 128-band hypercube. By distilling the data into two primary components capturing over 92% of the cumulative variance, the framework establishes a “spectrochemical bridge” that isolates hidden chromatic and biochemical variances invisible to traditional RGB sensors. This high-significance feature space allows the SVM to overcome the “Euclidean trap” inherent in unsupervised clustering, which frequently suffers from “class collapse” in roasted materials. Experimental results demonstrate that the hierarchical pipeline achieves an optimal overall accuracy of 88.6% and a Kappa coefficient of 0.378. The system attained high reliability during the Stage-1 binary screening, achieving an F1-score of 0.922 to protect the primary coffee matrix, while the Stage-2 multi-class model successfully mapped the spatial distribution of the highly camouflaged allergens. By providing pixel-wise, automated risk assessments, this work establishes a data-driven proof-of-concept for ‘Smart Food Safety’ systems, highlighting the potential for forensic authentication in future industrial quality control environments.

## Introduction

Coffee stands as one of the pillars of the modern global economy, ranking as the second most-traded commodity worldwide after petroleum^1^. It is the primary source of income for over 25 million farming families across the equatorial belt, particularly in developing nations such as Brazil, Vietnam, and Colombia^2^. However, the global coffee supply chain is currently facing a convergence of challenges. Rising production costs, labor shortages, and the increasing frequency of adverse climatic events, driven by global climate change, have led to significant volatility in coffee bean prices^3,4^. In this high-stakes economic environment, coffee has become a prime target for Economically Motivated Adulteration (EMA)^5^. Unscrupulous actors often seek to maximize profit margins by diluting high-value ground coffee with lower-cost substitutes^6^. While the substitution of Arabica beans with cheaper Robusta varieties is common, a more concerning practice involves the incorporation of non-coffee biological fillers. These adulterants, often by-products of other agricultural industries, include barley, soybeans, spent coffee grounds and dateseeds^7–9^. This fraudulent practice not only undermines the market integrity and erodes consumer trust but also presents a complex challenge for regulatory bodies attempting to verify the authenticity of processed powders^10^. While the economic implications of adulteration are severe, the public health risks associated with undeclared contaminants are paramount. The transition from whole beans to ground powder masks the physical identity of impurities, making visual detection impossible for the consumer^11^. Crucially, many common adulterants act as potent allergens. For instance, Soybeans are classified as a major food allergen, capable of triggering anaphylaxis in sensitized individuals^12,13^. Similarly, Barley contains gluten, posing a severe health threat to individuals with Celiac disease or gluten intolerance^14^. Conversely, adulterants like Dateseeds or coffee husks, while generally non-toxic, represent a fraudulent reduction in nutritional value and flavor quality^15^. Therefore, the industrial requirement for quality control has evolved. It is no longer sufficient to simply detect that a sample is adulterated (anomaly detection); modern food safety systems must be capable of identifying exactly what the contaminant is (forensic classification) to distinguish between a harmless filler and a dangerous allergen^16^. This necessity for specific identification raises the technological bar significantly, requiring analytical methods capable of untangling the complex chemical signatures of mixed organic powders^17^. Historically, the verification of coffee authenticity has relied on a suite of traditional analytical methods, each with inherent limitations that hinder their deployment in high-throughput industrial environments.

- Sensory analysis: Cupping and sensory evaluation, while the gold standard for flavor profiling, are subjective, non-scalable, and incapable of detecting low-level adulteration or identifying odorless contaminants^18,19^.
- Microscopy: Morphological analysis via microscopy can identify foreign plant structures (e.g., starch granules), but it is labor-intensive, time-consuming, and requires highly skilled technicians to manually inspect samples^20,21^.
- Wet chemistry & chromatography: Advanced chemical techniques such as High-Performance Liquid Chromatography (HPLC) and Gas Chromatography-Mass Spectrometry (GC-MS) offer high sensitivity for specific chemical markers^22–24^. However, these methods are destructive, expensive, and involve complex sample preparation using hazardous reagents^25^.
- DNA analysis: Polymerase Chain Reaction (PCR) techniques are highly specific for biological identification but are slow and costly, making them unsuitable for real-time monitoring^26,27^.

These “wet-lab” approaches share a common bottleneck: they are offline, batch-oriented processes that cannot keep pace with the speed of modern manufacturing lines^28^. Consequently, the industry has actively sought rapid, non-destructive, and eco-friendly alternatives^29^. Optical spectroscopy, particularly in the Near-Infrared (NIR) and Fourier Transform Infrared (FTIR) regions, has emerged as a leading solution for rapid food analysis^30–32^. These techniques analyze the interaction of light with molecular bonds (primarily C-H, O-H, and N-H) to generate a spectral fingerprint^33^. However, standard spectroscopic sensors typically function as “point detectors,” averaging the spectral signal over a given area^34^. In heterogeneous mixtures like adulterated coffee powder, this averaging effect is detrimental; the spectral signature of a small contaminant particle is often drowned out by the dominant coffee matrix, leading to false negatives^35,36^. Furthermore, the specific classification of roasted adulterants presents a unique chemical challenge. The roasting process induces the Maillard reaction and caramelization in all carbohydrate-rich grains (coffee, barley, soybeans, dateseeds), resulting in a convergence of physical color (brown) and chemical composition^37,38^. To a standard sensor, the spectral “distance” between roasted coffee and roasted barley is minimal^39^. Distinguishing these materials requires a system that can resolve both the spatial distribution of particles (to avoid averaging) and minute spectral variances (to distinguish chemical types)^40^. Hyperspectral Imaging overcomes this by capturing a “hypercube” (*x*, *y*, *λ*) of data^41,42^. Specifically, diffuse reflection HSI is uniquely suited for granular powders; unlike surface color imaging, it captures light scattered internally, revealing the chemical composition beneath the surface^43,44^. This capability has led to recent advances in grain quality assessment^45^ and soil analysis^46^. Several studies have successfully applied HSI to detect coffee adulterants, though they often focus on binary detection or specific fillers like husks^47–51^. However, effective multi-class identification relies heavily on the machine learning strategy employed. Despite the capabilities of HSI, existing research often neglects the forensic necessity of identifying specific allergens within spectrally similar mixtures. Furthermore, there is a lack of comparative studies evaluating how supervised versus unsupervised learning strategies handle the complex spectral overlap of roasted grains. To contextualize the necessity of this forensic approach, Table 1 presents a comparative analysis of recent studies that utilize HS imaging and advanced spectroscopy for coffee authentication. Existing literature rarely addresses the multi-class forensic isolation of specific high-risk allergens (e.g., soybeans and barley) from within the challenging environment of the Maillard convergence.

**Table 1:** Comparison of recent HS and advanced spectroscopic studies for coffee adulteration detection.

Reference	Imaging / Spectral Range	Target Adulterants	Analytical / ML Algorithm	Identified Limitation / Gap
Munyendo et al. (2024)^9^	NIR Spectroscopy	Chicory, robusta	Partial least square (PLS) regression	Point-based detection; lacks pixel-wise spatial mapping of contaminants.
Xie & Tan (2022)^31^	Front-face Synchronous Fluorescence Spectroscopy	Maize and soybean flours in Arabica coffee	Chemometric classification	Relies on averaged emission spectra over a fixed area; cannot perform pixel-wise forensic isolation of distinct biological allergens.
Yulia et al. (2023)^32^	Portable LED-based fluorescence spectroscopy	Green coffee beans (Authentication of cherry processing methods)	Chemometric analysis	Evaluates unroasted (green) whole beans; does not address the complex thermal spectral overlaps (Maillard convergence) induced during roasting and grinding.
Zhang et al. (2025)^39^	NIR Spectroscopy	General coffee powder adulteration	Pattern recognition / Chemometrics	Utilizes portable point-scans which capture bulk sample properties; cannot physically localize or isolate discrete, camouflaged allergen particles.
Zheng & Kamruzzaman (2024)^43^	HSI	General HSI applications in the coffee industry	Various machine learning models	Notes that while HSI is promising, existing applications largely focus on binary detection or bulk properties, lacking pixel-wise, multiclass forensic frameworks for heavily roasted matrices.
Khodabakhshian et al. (2026)^48^	FTIR	General adulteration	Pattern recognition	Destructive/offline setup; lacks spatial resolution for high-throughput screening.

In this study, we propose a hierarchical, two-stage forensic detection framework for coffee adulteration using diffuse reflection HSI. We specifically target three common biological adulterants, namely Barley (Gluten), Soybeans (Allergen), and Dateseeds (Organic Waste), and provide a deep comparative analysis of supervised SVM versus unsupervised K-Means approaches. Our specific contributions are:

- Refined spatio-spectral data acquisition: A rigorous data extraction protocol using expanded 40 × 40-pixel regions of interest (ROIs) and a spectral quality control filter were implemented that rejects any samples with a coefficient of variation (CV) exceeding 0.10. Furthermore, we utilize a multi-source annotation strategy involving interactive polygon labeling to generate high-fidelity ground-truth maps for precise pixel-wise evaluation.
- Interpretability-driven dimensionality reduction (PCA): A strategic dimensionality reduction protocol was implemented using PCA to resolve spectral redundancy across the 128-band hypercube. We demonstrate that the first two principal components capture over 92% of the total cumulative variance, distilling complex data into a high-significance feature space. This establishes a “spectrochemical bridge” where PC1 and PC2 are explicitly linked to biochemical structural variance and roasting-induced chromatic variations, respectively, ensuring model robustness and scientific interpretability.
- Two-stage hierarchical strategy: A cascaded classification pipeline was introduced. Stage 1 performs binary discrimination (coffee vs. non-coffee) to rapidly flag anomalies, while Stage 2 executes multi-class identification on the identified “non-coffee” pixels to pinpoint the specific contaminant type (barley, soybeans, or dateseeds).
- Quantified comparative methodology (SVM vs. K-Means): A definitive performance benchmark between supervised SVM and unsupervised K-Means paradigms were provided. We demonstrate that the hierarchical SVM is the optimal performing model, achieving an 88.6% OA. In contrast, we show that even with a two-stage architecture, unsupervised clustering suffers from “class collapse” and fails to detect specific fillers like dateseeds.
- Forensic identification of allergens: We demonstrate that the proposed framework successfully disentangles overlapping spectral signatures to provide the specific identification required for modern food safety. Our proposed results show balanced improvements across all adulterant classes, ensuring that high-risk allergens are reliably unmasked.

While the foundational chemometric operators utilized in this pipeline (e.g., PCA, SVM) are established, the methodological innovation of this work lies in their novel architectural integration. Conventional “flat” classification approaches frequently fail under the extreme non-linear overlaps of roasted organics. To overcome this limitation, this study introduces a decoupled, two-stage hierarchical framework. By specifically engineering this pipeline to isolate intrinsic biochemical markers from roasting-induced thermal variance, this architecture successfully translates established bulk-spectroscopy tools into a spatially resolved, multi-class forensic diagnostic system capable of breaking the Maillard convergence. This proposed work establishes a data-driven foundation for “Smart Food Safety” systems, proving that supervised learning is indispensable for the forensic differentiation of chemically similar biological contaminants.

## Materials and methods

### Sample preparation

**Fig. 1:**
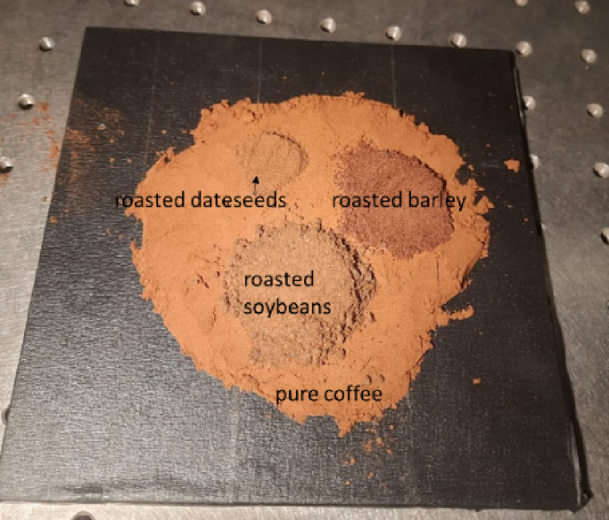
Materials used in this study for comparative analysis, including pure Arabica coffee and three biological adulterants: roasted barley, roasted dateseeds, and roasted soybeans.

The study utilized four distinct experimental scenes (S0–S3) designed to simulate realistic forensic scenarios of EMA. The primary matrix consisted of 100% pure Arabica coffee beans, established as the high-value target. Three spectrally challenging biological adulterants were selected based on their prevalence in fraud cases and their specific chemical implications: roasted barley (representing gluten contamination), roasted soybeans (representing allergenic hazards), and roasted dateseeds (representing non-toxic organic waste fillers), as depicted in Fig. 1. To guarantee commercial authenticity and regional relevance, all four material species were procured in their roasted forms from established commercial vendors and retail markets in Cairo, Egypt. Crucially, to establish an unadulterated baseline and eliminate any risk of pre-existing commercial contamination, the premium Arabica coffee beans were verified and ground under direct supervision at the point of procurement, guaranteeing absolute matrix purity and the accuracy of the baseline reference spectra. To ensure the HS classification challenge relied solely on chemical composition rather than physical morphology, strict sample standardization was applied. The commercially sourced roasted materials were selected based on their matched medium-dark roast levels, which exhibit the advanced Maillard reaction and caramelization features responsible for the convergence of their visible color profiles. Subsequently, the roasted adulterant samples were ground and sieved in the laboratory, and all material powders were strictly monitored to achieve a homogeneous particle size distribution, mimicking the texture of commercial ground coffee. The powdered samples were arranged on matte black, non-reflective trays to minimize background specular reflection. The scenes were constructed as follows:

- Scene S0 (Reference): Contained 100% pure Arabica coffee to establish a baseline for false-positive assessment.
- Scenes S1–S3 (Adulterated): Contained controlled, spatially distinct regions of pure coffee alongside discrete patches of barley, soybeans, and dateseeds. This arrangement created a “ground truth” layout with unambiguous boundaries, facilitating precise pixel-wise validation.

As shown in Fig. 1, the samples were leveled to form optically opaque layers (approx. 5 mm depth), ensuring that the recorded signal originated entirely from diffuse reflectance within the sample volume rather than the underlying substrate. From these constructed scenes, data volume was quantified by extracting five distinct spatial replicates per material class, providing a statistically rigorous foundation for subsequent proposed chemometric and machine learning evaluation.

### Hyperspectral imaging system and instrumentation

**Fig. 2:**
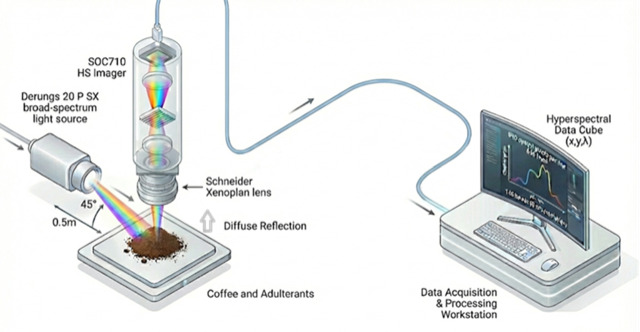
Schematic diagram of the HS image acquisition system. The setup employs a push-broom SOC710 HS Imager (Surface Optics Corp.) positioned at a 0° nadir angle, equipped with a Schneider Xenoplan lens (35 mm, F/1.9) for high spatial resolution. Diffuse reflectance is captured using a Derungs 20 P SX broad-spectrum light source oriented at a 45° angle of incidence. The internal optical assembly (shown in cutaway) directs incoming light through a slit and collimating optics before a transmission diffraction grating disperses it onto the CCD sensor. This configuration generates a continuous high-fidelity spatial-spectral datacube (x, y, λ). covering the VIS–NIR range (400–1000 nm).

The acquisition of high-dimensional spectral data was performed using a state-of-the-art push-broom scanning system designed for laboratory-grade diffuse reflectance measurements is shown in Fig. 2. The core of the system is the SOC710 HS Imager (Surface Optics Corp., USA), a radiometrically calibrated device capable of capturing simultaneous spatial and spectral information with high fidelity. The imager incorporates a high-sensitivity linear Charge-Coupled Device (CCD) array coupled with a transmission diffraction grating. This architecture allows for the precise dispersion of incoming light into constituent wavelengths, covering the Visible to Near-Infrared (VIS–NIR) range from 400 to 1000 nm (0.4–1.0 μm). The system operates with a spectral resolution of approximately 5 nm, generating a continuous spectral signature across 128 contiguous bands for every spatial pixel, resulting in a dense spatial-spectral datacube *I* (*x*, *y*, *λ*). To ensure high spatial resolving power, the sensor was fitted with a Schneider Xenoplan lens (F/1.9, 35 mm focal length) providing a 10° Field of View (FOV). The system was configured to resolve fine granular details with a manufacturer-specified nominal spatial resolution of < 40 μm, capturing 520 spatial pixels per line. A typical scan for this study comprised an average of 696 lines, capturing the complete morphological and chemical distribution of the coffee mixtures. Uniform excitation of the samples was achieved using a Derungs 20 P SX broad-spectrum light source (Derungs, Germany), selected for its high radiometric homogeneity across the 0.4–1.0 μm range. To strictly enforce a diffuse-reflectance collection geometry and eliminate specular glare from the granule surfaces, the illumination source was positioned at a distance of 0.5 m and oriented at a 45° angle of incidence relative to the sample plane. The HS camera was mounted at a 0° (nadir) angle, ensuring that the recorded signal consisted primarily of internally scattered light carrying the chemical absorption features of the coffee and adulterants.

To ensure statistical significance and experimental reproducibility, a structured sampling and replication protocol was implemented. A total of four primary experimental scenes (S0–S3) were constructed to provide a comprehensive baseline and varied adulteration scenarios. For each of the four material classes (pure Arabica coffee, roasted barley, roasted soybeans, and roasted dateseeds), five independent 40 × 40-pixel ROIs were extracted from the HS data cubes to serve as spatial replicates. This spatial sampling strategy initially targeted 8,000 spectral pixels (computational samples) per material class (1,600 pixels per ROI × 5 replicates). Following the spectral quality control step, the retained pixel counts after outlier trimming were strictly consistent across all four classes: barley (7,944 pixels), coffee (7,944 pixels), dateseeds (7,944 pixels), and soybeans (7,944 pixels). Because these retained counts were naturally equal, a direct result of standardizing the number of extracted ROIs and spatial patch sizes per class, no additional mathematical down-sampling was required to achieve class balance. By enforcing this strict 1:1:1:1 numerical parity, the framework generated a perfectly balanced final training dataset comprising 31,776-pixel spectra across the four classes. This proactive balancing explicitly mitigates the risk of category imbalance, ensuring that the subsequent machine learning models and the reported macro-averaged metrics reflect genuine per-class discriminative performance rather than an artificial bias toward the dominant coffee matrix. Consequently, for the evaluation phase, the full experimental testing mixtures (including S0–S3 and the multi-contaminant scene) were deliberately prepared as random, macroscopic heterogeneous scatterings rather than fixed gravimetric weight percentages. The objective of these scenes is to validate the model’s forensic capacity to spatially isolate specific, camouflaged pixels against a dominant coffee background, regardless of the overall bulk concentration. Furthermore, the generalizability of the proposed framework was tested using an independent, blind test sample containing a coffee-barley mixture, acting as an external validation replicate to ensure the model’s performance was not scene-dependent. The final pixel-wise evaluation was performed against high-fidelity ground-truth maps covering the full spatial extent of the experimental scenes, ensuring that the reported accuracy reflects thousands of independent spectral measurements.

### Image acquisition and radiometric correction

HS data acquisition was conducted under controlled laboratory conditions to ensure high signal-to-noise ratio (SNR) and radiometric consistency. The SOC710 imager was allowed to warm up for 30 min prior to scanning to stabilize the CCD sensor temperature and minimize dark current drift. For each scene, raw intensity data (*I*_*raw*_) were captured as a 12-bit digital number hypercube. To account for the system’s dark current noise and the non-uniform spatial distribution of the illumination source, a standard white-dark radiometric calibration was applied to convert raw intensity into reflectance units. Two reference standards were acquired prior to sample scanning:

- Dark reference (*I*_*dark*_): Obtained by closing the camera’s mechanical shutter (0% reflectance) to capture the sensor’s inherent thermal noise.
- White Reference (*I*_*white*_): Obtained by scanning a certified Spectralon^®^ panel (99% reflectance) under identical illumination conditions to characterize the incident light profile.

The relative reflectance spectrum (*R*) for each pixel (*x*, *y*) at wavelength *λ* was calculated using Eq. (1)^52^:

1 $$\:R\left(x,\:y,\lambda\:\right)=\frac{{I}_{raw}\left(x,\:y,\lambda\:\right)-{I}_{dark}\left(x,\:y,\lambda\:\right)}{{I}_{white}\left(x,\:y,\lambda\:\right)-{I}_{dark}\left(x,\:y,\lambda\:\right)}$$

This step effectively removed systematic errors and corrected for sensor pixel response non-uniformity.

### Spectral preprocessing pipeline

Following radiometric calibration, the reflectance data contained inevitable high-frequency noise and scattering effects caused by the granular surface texture of the coffee powder. To maximize signal fidelity and isolate chemical features, a dedicated preprocessing and quality control pipeline was implemented:

- Spectral smoothing (Savitzky-Golay Filter): A Savitzky-Golay filter (2nd order polynomial, 15-point window) was applied to the spectral dimension of each pixel. This technique suppresses random high-frequency noise while preserving the shape and height of specific absorption peaks critical for discriminating between roasted biological materials.
- Standard normal variate (SNV) transformation: To mitigate multiplicative scaling effects caused by sample surface roughness and light scattering (physical path length differences), SNV normalization was performed on each pixel spectrum. Each spectrum *x*_*i*_ was centered and scaled according to Eq. (2)^53^:

2 $$\:{x}_{SNV}=\frac{{x}_{i}-{\mu\:}_{i}}{{\sigma\:}_{i}}$$

where *µ*_*i*_ is the mean intensity and $\:{\sigma\:}_{i}\:$is the standard deviation of the spectrum *i*. This transformation aligns the baseline and intensity scale of all pixels, ensuring models focus on chemical spectral shapes rather than absolute intensity variations due to particle size or lighting geometry.

- Spectral quality control: To ensure the purity of the training data, an automated quality control filter was implemented during data extraction. Any extracted ROI exhibiting a high CV > 0.10 in its spectral response was systematically rejected. This step ensures that only spectrally consistent and representative pixels are utilized for model training, reducing the influence of outliers or edge-related artifacts.

### Principal component analysis (PCA) for feature extraction

To address the inherent spectral redundancy of the 128-band HS cube and mitigate the “curse of dimensionality,” PCA was integrated into the computational pipeline. In diffuse-reflectance HSI, adjacent spectral bands are highly correlated, often providing redundant information regarding the same molecular vibrations or electronic transitions. This high-dimensional complexity can lead to overfitting in supervised models and increase the computational cost of pixel-wise classification.

The PCA algorithm transforms the original correlated variables into a new set of orthogonal, uncorrelated variables termed Principal Components (PCs). This transformation is achieved through an eigenvalue decomposition of the spectral covariance matrix, where the resulting components are ordered by the amount of total variance they capture from the original dataset. For this forensic framework, PCA serves two critical functions beyond mere data compression:

- Spectral de-noising: By retaining only the top-tier components that capture the majority of the cumulative variance, the framework effectively filters out high-frequency sensor noise and minor illumination fluctuations that often occupy lower-order components.
- Feature interpretability: The loading weights of the first two components provide a “spectrochemical bridge” for the model. PC1 is primarily influenced by broader biochemical features across the near-infrared plateau (600–1000 nm), such as lipid and carbohydrate variations. Conversely, PC2 captures high variability in the visible region (400–600 nm), specifically targeting the pigment variations induced by the Maillard reaction during roasting.

PCA was employed as a strategic marker-selection protocol. Instead of treating the 128 bands as generic image features, the loading weights were analyzed to identify spectroscopic markers associated with known biochemical constituents (lipids/carbohydrates) and thermal indicators (melanoidins).This reduced feature space provides the SVM with a simplified, high-significance input, allowing the Radial Basis Function (RBF) kernel to construct more robust and generalizable non-linear decision boundaries between the coffee matrix and the camouflaged adulterants.

### Region of Interest (ROI) selection and ground-truth annotation

**Fig. 3:**
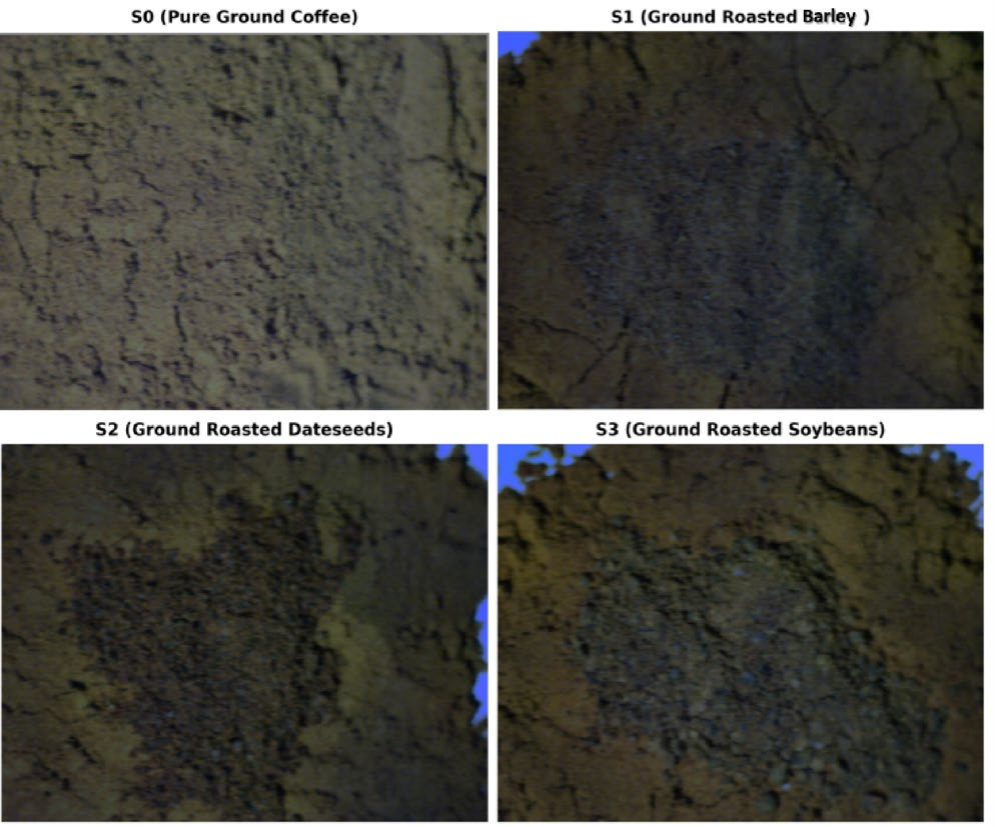
The true-color RGB renderings from selected HS scenes (S0–S3) used to simulate EMA in ground coffee. Scene S0 contains 100% pure Arabica coffee, while scenes S1–S3 include spatially distinct regions of coffee adulterated with roasted barley, dateseeds, and soybeans. All materials were roasted, ground, and sieved to a uniform particle size and arranged on a matte, non-reflective background to minimize morphological and background effects.

Regions of interest were manually delineated on true-color composite images using interactive annotation tools, guided by a priori knowledge of the sample composition and the visually discriminable morphological features of the constituent materials. As shown in Fig. 3, the medium-dark roasting process induces a visual convergence that renders the adulterants almost indistinguishable from the coffee matrix. All materials were sieved to a uniform particle size and arranged on a matte, non-reflective background to minimize morphological artifacts.

**Fig. 4:**
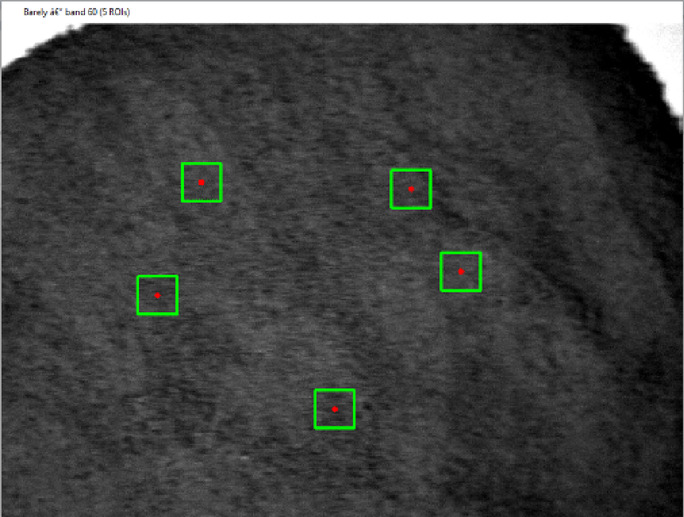
Representative visualization of the interactive selection of five distinct 40 × 40-pixel ROIs from a roasted barley sample data cube at band 60.

The selection process adhered to a rigorous protocol to maximize spectral purity and representativeness:

- Expanded ROI extraction and quality control: An interactive OpenCV-based tool was used to select five distinct ROI centers per sample. To capture a broader range of the material’s natural intrinsic variability, including variations in particle size and surface scattering, the ROI size was increased to 40 × 40 pixels. To ensure training data integrity, an automated filter was implemented to reject any ROI exhibiting a CV > 0.10. This protocol, illustrated in Fig. 4, ensures the model is conditioned on the material’s chemical signature rather than localized lighting artifacts or outliers.

- Class balancing and spatial partitioning: The final extracted dataset comprised the strictly balanced, quality-controlled spectra for the four material classes (pure coffee, barley, soybeans, and dateseeds). Crucially, to explicitly prevent spatial data leakage and the artificial overestimation of model performance, all data partitioning was executed strictly at the spatial ROI level, rather than via random pixel-level splitting. Because adjacent pixels within a physical powder patch exhibit high spatial autocorrelation, a randomized pixel-level split would inevitably contaminate the validation data with target signatures already seen during training. By ensuring that the entire, spatially distinct 40 × 40-pixel ROIs were kept intact and strictly isolated during the subsequent *GroupKFold* cross-validation phase, the framework was forced to learn generalized chemical markers rather than memorizing localized spatial artifacts.

To facilitate rigorous pixel-wise evaluation across the complete experimental scenes (S0–S3), high-fidelity ground-truth label maps were generated using an interactive polygon labeling strategy. This approach integrated spatial prior knowledge, high-resolution RGB inspection, and spectral validation to assign class indices: 0 (barley), 1 (coffee), 2 (dateseeds), 3 (soybeans), and 4 (background).

**Fig. 5:**
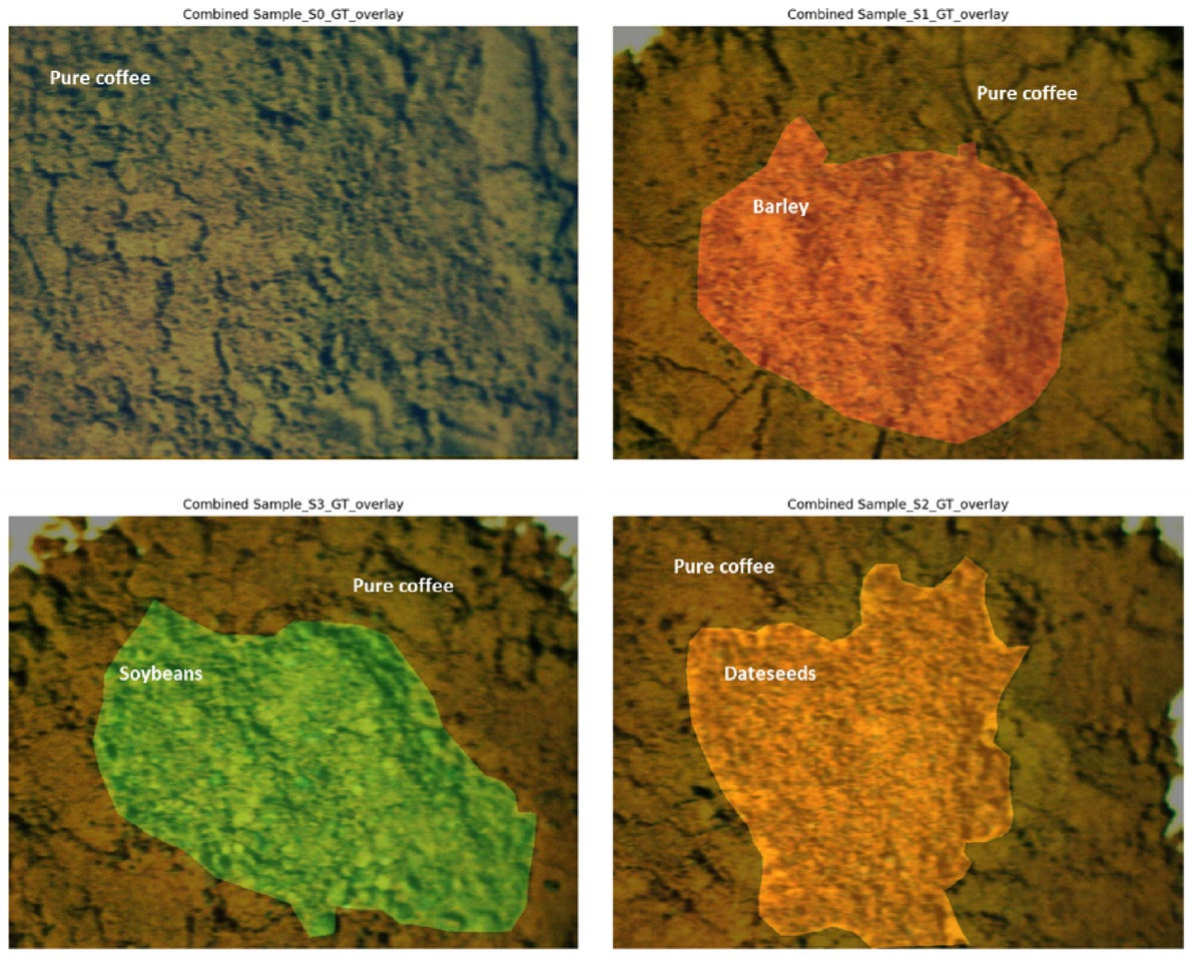
High-fidelity ground-truth label maps generated via interactive polygon labeling for scenes S0–S3. Colored overlays represent the gold-standard regions used for pixel-wise performance validation.

- Boundary exclusion protocol: A critical challenge in granular powder analysis is the presence of “mixed pixels” at physical interfaces. To prevent these ambiguous data points from introducing labeling noise, a boundary exclusion protocol was implemented, as described in Fig. 5. Optically ambiguous edges were assigned to an “Ignore” class (Mask Value = 255) and systematically excluded from the quantitative confusion matrix.

- Justification of the “Ignore” class: Regarding the forensic challenge of “Maillard Convergence,” the primary objective of this study is to benchmark the system’s ability to resolve chemical spectral overlap. Boundary pixels often represent non-representative spectra resulting from physical shadowing or mechanical mixing (morphological noise) rather than pure chemical signatures. Excluding these pixels ensures that the proposed reported accuracy reflects the model’s spectrochemical discrimination power without being confounded by physical edge artifacts. While these regions are excluded from quantitative metrics to maintain labeling purity, they remain present in the final classification maps to demonstrate the system’s spatial coherence in real-world scenarios.

### Machine learning algorithms

To evaluate the comparative efficacy of unsupervised versus supervised approaches in resolving the spectral overlap of roasted powders, two distinct machine learning frameworks were implemented: K-Means clustering (unsupervised) and SVM (supervised).

#### Unsupervised approach: K-means clustering

The K-Means algorithm was employed as a baseline method to test the separability of the data without prior labeling. This iterative algorithm partitions the spectral dataset *X* into *K* distinct, non-overlapping clusters by minimizing the within-cluster sum of squares (WCSS)^54^.

3 $$\:J=\sum\:_{j=1}^{K}\sum\:_{i=1}^{n}{\lVert{x}_{i}^{\left(j\right)}-{\mu\:}_{j}\lVert}^{2}$$

where *µ*_j_ represents the centroid of cluster *j*. As an unsupervised reference method, K-means clustering was applied to the same preprocessed spectral data to provide a baseline for comparison with supervised approaches. The number of clusters was selected based on the expected number of dominant spectral classes. For binary segmentation, *K* = 2 was employed to approximate the separation between coffee and non-coffee materials. For multiclass segmentation, including background contributions, *K* = 4 was used to represent coffee, adulterant materials, and the tray background.

#### Supervised approach: Support vector machine (SVM)

To overcome the limitations of linear clustering, a SVM classification framework was developed. SVM is a robust supervised learning model that constructs an optimal decision boundary (hyperplane) that maximizes the margin between classes^55^. Given the inherent non-linearity of biological spectral data, where roasted grains exhibit similar overlapping signatures, we utilized the Kernel Trick to project the data into a higher-dimensional space. Specifically, the RBF kernel was employed, defined mathematically as^56,57^:

4 $$\:K\left({z}_{i},{z}_{j}\right)=exp\left(-\gamma\:{\lVert{z}_{i}-{z}_{j}\lVert}^{2}\right)$$

**Fig. 6:**
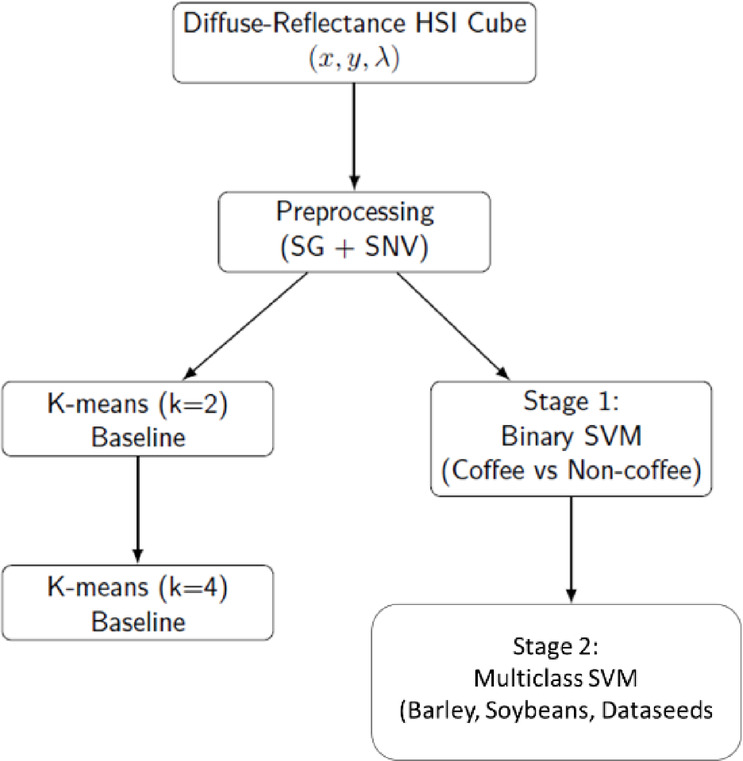
Schematic flowchart of the comparative computational framework employed for forensic coffee analysis. Phase I (Preprocessing): Raw diffuse-reflectance datacubes (x, y, λ) undergo spectral smoothing (Savitzky-Golay, SG) and scatter correction (Standard Normal Variate, SNV) to maximize signal fidelity. Phase II (Comparative Classification): The pipeline splits to evaluate two distinct strategies: (Left) Unsupervised Baseline: A K-Means clustering approach tested at both binary (K = 2) and multi-class (K = 4) levels to assess spontaneous spectral separability. (Right) Proposed Hierarchical System: A supervised Two-Stage SVM framework designed for forensic precision. Stage 1 performs rapid binary screening to flag anomalies (coffee vs. non-coffee), while Stage 2 executes specific multi-class identification to discriminate between biological adulterants (barley, soybeans, dateseeds).

Where $\:K\left({z}_{i},{z}_{j}\right)$ is the kernel function computing the similarity between two spectral vectors, z_i_ and *z*_*j*_ represent the feature vectors in the reduced PCA space (comprising PC1 and PC2 scores), $\:{\lVert{z}_{i}-{z}_{j}\lVert}^{2}$ is the squared Euclidean distance between the two spectra, and $\:\gamma\:$ is a tunable hyperparameter that controls the influence of a single training example. Two-stage hierarchical strategy:

- Stage 1 (Binary screening): A binary SVM model was first trained to distinguish “pure coffee” (target) from “non-coffee” (anomaly). This acts as a rapid quality control filter.
- Stage 2 (Forensic identification): Pixels flagged as “non-coffee” were passed to a multi-class SVM model trained to specifically identify the contaminant type (barley vs. soybean vs. dateseed). This hierarchical structure mirrors the industrial need for first flagging a problem, and then diagnosing it. Figure 6 summarizes the overall diffuse-reflectance HSI acquisition and two-stage SVM pipeline.

### Hyperparameter optimization

All computational procedures, including spectral preprocessing, dimensionality reduction (PCA), model training, and quantitative evaluation, were implemented in Python (version 3.12.7) operating within a 64-bit (AMD64) Anaconda distribution environment. The core machine learning and cross-validation architectures were executed utilizing the *scikit-learn* library (version 1.5). This pipeline was computationally supported by *NumPy* and *SciPy* for high-dimensional matrix operations, *OpenCV* for the extraction of spatial ROIs, and *Matplotlib* for data visualization. The classification framework was executed using the SVM algorithm with the RBF kernel, specifically selected for its ability to map the non-linearly separable spectral data of roasted grains into a higher-dimensional feature space. To optimize the model’s generalization capability and mitigate the risk of overfitting, the critical hyperparameters were tuned using an exhaustive grid search. Crucially, to strictly prevent spatial data leakage between adjacent pixels originating from the same physical powder patch, cross-validation was conducted utilizing a *GroupKFold* strategy (k = 5), with the ROI spatial identity acting as the grouping variable. The optimization metric was set to the macro-averaged F1-score rather than simple accuracy to ensure the model prioritized the correct identification of minority classes. The exhaustive grid search was conducted over the following parameter space, yielding 27 candidate combinations per stage (27 fits × 5 folds = 135 total fits per stage):

- Regularization penalty (*C*): Searched over {10, 30, 100}.
- Kernel coefficient ($\:\gamma\:$ ): Searched over {‘scale’, 0.01, 0.03}.
- PCA variance retention: Searched over {0.95, 0.98, 0.99}.

For both Stage 1 (binary coffee vs. non-coffee screening) and Stage 2 (multi-class adulterant identification), the grid search selected identical optimal parameters: *C* = 10, and a PCA retention threshold of 99%. Furthermore, this adaptive formulation yielded an exact numeric value of $\:\gamma\:$ = 0.007596, derived directly from the 51 PCA components retained at the 99% variance threshold and a scaled training feature variance of 2.581. This adaptive scaling ensures the RBF kernel width scales optimally with the dimensionality of the reduced feature space. These optimized models were subsequently locked and deployed for the final spatial classification of the unseen test scenes.

## Experimental results and analysis

### Principal component analysis (PCA) for spectral redundancy and interpretability

To address the high dimensionality and significant spectral redundancy inherent in the 128-band HS datacubes, PCA was performed on the preprocessed reflectance spectra. This step simplifies the subsequent classification modeling while improving both computational efficiency and model robustness.

#### Analysis of spectral redundancy (Scree Plot)

**Fig. 7:**
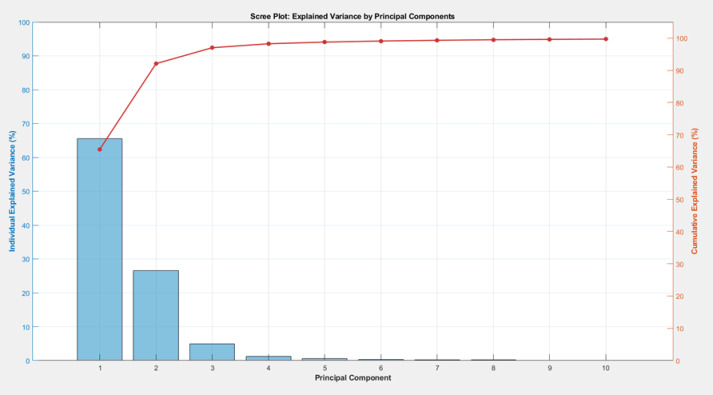
Scree plot showing individual percentage of variance (bars) and cumulative explained variance (line) for the roasted coffee and adulterant spectra.

The Scree Plot (Fig. 7) illustrates the individual and cumulative variance explained by the first ten principal components. The analysis reveals that the vast majority of spectral information is concentrated in the first few components. Specifically, PC1 captures 65.5% of the total variance, while PC2 accounts for 26.6%. Together, these two components successfully explain over 92% of the total cumulative variance. The sharp “elbow” observed after the second component confirms that the primary chemical and physical differences between roasted coffee and its adulterants can be captured in a significantly reduced feature space.

#### Spectral interpretability (Loadings Plot)

**Fig. 8:**
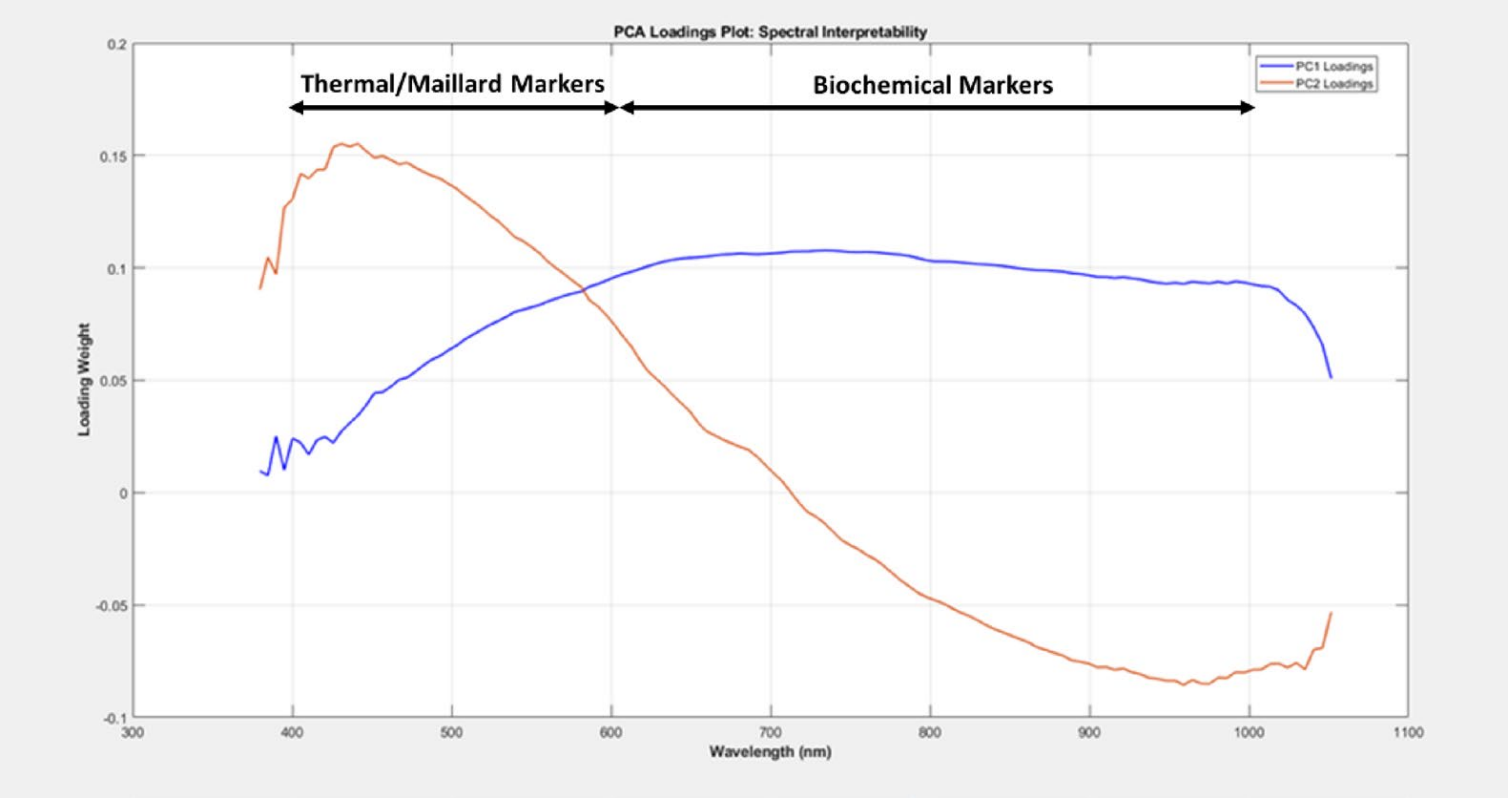
PCA loadings plot illustrating the contribution (loading weights) of individual wavelengths for the first two principal components.

To refute concerns regarding the purely computational nature of these reduced features, a PCA Loadings Plot (Fig. 8) was utilized to identify the specific spectral regions driving the variance. This aligns the mathematical reduction with known biochemical distinctions.

**-** PC2 (Visible sensitivity): PC2 (orange line) exhibits high absolute loading weights and significant variability across the visible spectral range (400–600 nm). This indicates a heightened sensitivity to pigment-related differences and color variations induced by the Maillard reaction.

PC1 (Biochemical influence): PC1 (blue line) demonstrates broader contributions across the near-infrared plateau (approximately 600–1000 nm). These loading weights suggest a primary influence from diverse biochemical components, such as lipids or carbohydrates, which remain distinct across the different sample types despite visual similarity.

To fulfill the requirement for chemical marker data, the PCA loadings (Fig. 8) were analyzed to identify specific wavelength regions driving the discrimination:

- PC2 (Visible/Thermal sensitivity): PC2 exhibits high absolute loading weights and significant variability across the visible spectral range (400–600 nm). While definitive chemical identification requires independent analytical validation, this mathematical variance is highly consistent with established spectroscopic literature, which identifies this visible region as heavily influenced by melanoidins and browning pigments formed during the Maillard reaction^58,59^.
- PC1 (Biochemical/Structural influence): PC1 demonstrates broader contributions across the near-infrared plateau (approximately 600–1000 nm). These loading weights align with the well-documented vibrational overtones of C-H and O-H bonds^60,61^. Consequently, this component serves as a robust mathematical proxy for the underlying variance in structural biochemistry (such as lipid and carbohydrate profiles), which remains distinct across the different sample types despite their visual similarity.

By correlating mathematical variance with these known spectroscopic features, the proposed framework ensures that the classification is rooted in intrinsic chemical markers rather than superficial image artifacts.

#### PCA dimensionality reduction summary

To synthesize the findings from the Scree and Loadings analyses, Table 2 provides a comprehensive overview of the dimensionality reduction achieved. By concentrating the vast majority of spectral information into two primary components, the framework resolves the high dimensionality of the 128-band data cube into a localized, high-significance feature space suitable for robust machine learning.

**Table 2:** Summary of the first two principal components capturing the primary spectral variance in roasted coffee and adulterant samples.

Principal Component	Individual Variance (%)	Cumulative Variance (%)	Dominant Range (nm)	Chemical Marker Influence
PC1	65.5%	65.5%	600–1000 nm	Lipid/Carbohydrate markers
PC2	26.6%	92.1%	400–600 nm	Thermal/Maillard markers

#### PCA score plot

**Fig. 9:**
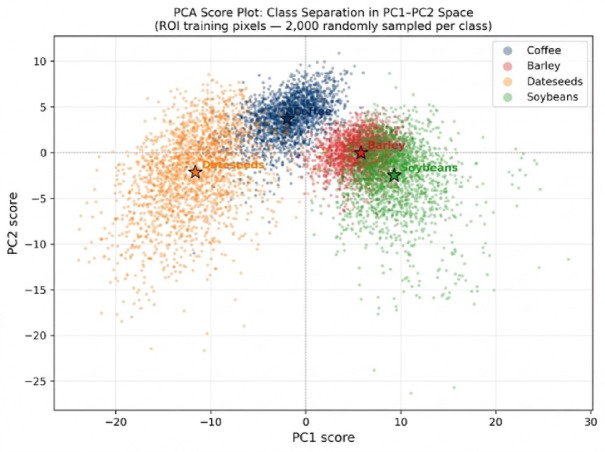
PCA score plot illustrating the distribution of ROI-derived training pixels in the reduced PC1–PC2 feature space for the four material classes: pure coffee, barley, dateseeds, and soybeans. For visual clarity, 2,000 pixels were randomly sampled from each class. Star markers indicate the respective class centroids. The plot demonstrates that PCA preserves meaningful class structure within the reduced feature space, while the partial overlap between clusters reflects the strong roasting-induced spectral similarity that necessitates advanced machine learning classification.

To complement the Scree and Loadings analyses, a PCA score plot was generated in the reduced PC1–PC2 feature space to visualize the distribution of the four material classes prior to SVM classification. As shown in Fig. 9, the ROI-derived training pixels form distinct, albeit partially overlapping, clusters. Pure coffee occupies a relatively compact, distinct region. In contrast, dateseeds are significantly shifted toward negative PC1 values, while roasted barley and soybeans are distributed toward positive PC1 values. The distinct centroid positions confirm that the reduced PCA space successfully preserves the fundamental, class-relevant biochemical structure. Furthermore, the observed overlap at the cluster boundaries visually reinforces the necessity of the subsequent non-linear SVM classifier to effectively resolve the strong spectral convergence induced by the roasting process.

### Analysis of diffuse reflectance response and spectral similarity

**Fig. 10:**
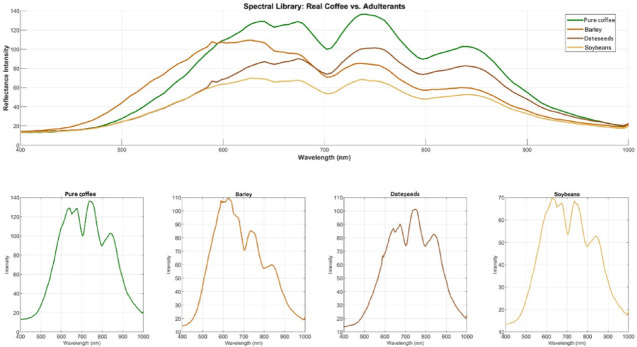
Spectral library and comparative reflectance analysis. (Top) Overlaid mean spectral signatures of pure Arabica coffee (Green) versus biological adulterants: Barley (Orange), Dateseeds (Brown), and Soybeans (Yellow) across the VIS–NIR range (400–1000 nm). (Bottom) Individual intensity profiles for each material class. Key observation: While the significant spectral overlap in the visible region (400–600 nm) illustrates the “Maillard Convergence,” PCA reveals that this region contains subtle chromatic variances (PC2, 26.6% of variance) that are invisible to the human eye but critical for forensic discrimination. When coupled with the biochemical signatures captured in the near-infrared plateau (PC1, 65.5% of variance), the framework achieves the high-dimensional resolution required to resolve the chemical identity of each mixture.

Figure 10 presents the mean spectral signatures of pure Arabica coffee alongside the three biological adulterants (barley, soybeans, and dateseeds) across the VIS–NIR range (400–1000 nm). Visually, all four classes exhibit a characteristic broad reflectance plateau between 600 and 900 nm, indicative of the browning pigments formed during HSI processing.

The “Maillard Convergence” phenomenon as illustrated in the spectral profiles, the roasting process induces a chemical convergence known as the Maillard reaction, where amino acids and reducing sugars condense to form melanoidins^62^. This reaction is responsible for the uniform brown coloration observed across all samples. Consequently, as seen in the 400–600 nm range, the reflectance intensities of barley and dateseeds overlap significantly with that of pure coffee. While these materials are visually indistinguishable to the human eye and standard RGB sensors, the implementation of PCA extracts the underlying variance (92.1% cumulative) that remains hidden within these overlapping profiles. This confirms that while conventional color imaging is inadequate, the hierarchical decoupling of spectral features into PC1 and PC2 allows the model to overcome the limitations of roasting-induced color similarity. This spectral similarity poses a fundamental challenge for unsupervised algorithms like K-Means.

- The Euclidean trap: K-Means operates by minimizing the Euclidean distance between data points and cluster centroids. As shown in Fig. 9, the spectral curves of the adulterants are highly collinear with Coffee. Without the non-linear feature mapping provided by the PCA-SVM pipeline, K-Means remains trapped by this intensity-based similarity, leading to the “class collapse” observed in the dateseed and soybean segments.
- Resulting confusion: Consequently, K-Means fails to detect a distinct “boundary” between these classes, often grouping them into a single “Roasted Material” cluster or segmenting them based on shadow intensity rather than chemical identity.

The data in Fig. 9 demonstrates that while the general intensity is similar, subtle absorption features remain distinct. These minute spectral nuances are effectively isolated through PCA, which summarizes the 128-band data into PC1 (biochemical structure) and PC2 (chromatic variance). By training the supervised SVM on this reduced, high-significance feature space rather than raw intensity magnitudes, the framework can construct the non-linear decision boundaries necessary for forensic identification, achieving a significant 88.6% overall accuracy.

### Stage-1 Binary screening: spatial coherence vs. intensity thresholding

**Fig. 11:**
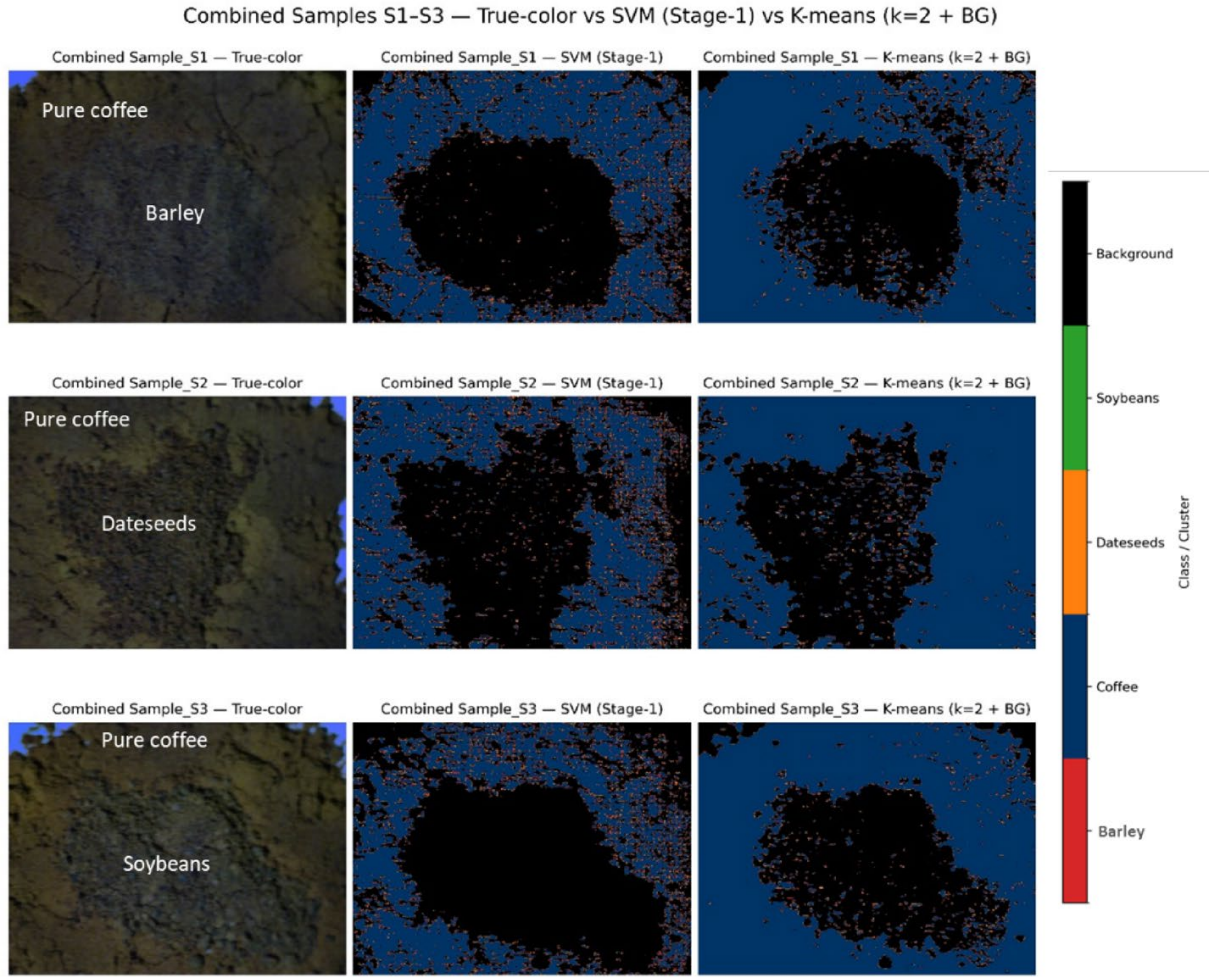
Comparative visualization of Stage-1 Binary screening (coffee vs. non-coffee) across mixed scenes S1 (barley), S2 (dateseeds), and S3 (soybeans). (Left) True-Color RGB: Reconstructed composites showing the visual ambiguity between the roasted coffee matrix and the adulterants. (Middle) Supervised SVM: The binary SVM classifier produces spatially coherent segmentation maps, accurately defining the morphological boundaries of the adulterant regions (rendered in Black) against the pure coffee background (rendered in Blue) with minimal noise. (Right) Unsupervised K-Means (K = 2): The clustering baseline exhibits significant “salt-and-pepper” noise and boundary fragmentation (visible as scattered blue pixels within the black adulterant regions), highlighting its sensitivity to intensity fluctuations rather than chemical spectral shape.

The performance of the proposed binary screening stage was evaluated by comparing the classification maps of the supervised SVM against the unsupervised K-Means baseline (K = 2). Figure 11 visually contrasts these outcomes across the mixed scenes (S1–S3).

Morphological fidelity of SVM as evidenced in the central column of Fig. 11, the SVM Stage-1 classifier demonstrates superior morphological fidelity. It accurately reconstructs the physical shape of the adulterant regions (depicted in black) against the coffee matrix (blue).

- Spatial consistency: The SVM masks exhibit high spatial coherence with sharp, continuous boundaries that closely match the true-color reference images.
- Noise suppression: The model effectively filters out intra-class variance, resulting in solid, uniform segmentation masks free from the “salt-and-pepper” noise often caused by particle shadowing.

The “Intensity Trap” of K-Means In contrast, the K-Means clustering results (right column) reveal the inherent limitations of unsupervised distance-based learning. While K-Means successfully identifies the core regions of the adulterants, the resulting masks are noticeably fragmented and eroded.

- In scene S2 (Dateseeds), the K-Means mask contains numerous misclassified “coffee” pixels (blue speckles) within the adulterant region. This suggests that the algorithm is clustering based primarily on pixel intensity rather than spectral shape. Lighter-colored particles of the adulterant are erroneously grouped with the coffee, while darker shadows in the coffee are grouped with the adulterant.
- Consequently, while K-Means can serve as a rough, label-free estimator of contamination, it lacks the chemical specificity required to define the precise spatial extent of the hazard, necessitating the use of the supervised SVM for accurate boundary delineation.

### Stage-2 Multiclass forensic identification: spectrochemical discrimination

**Fig. 12:**
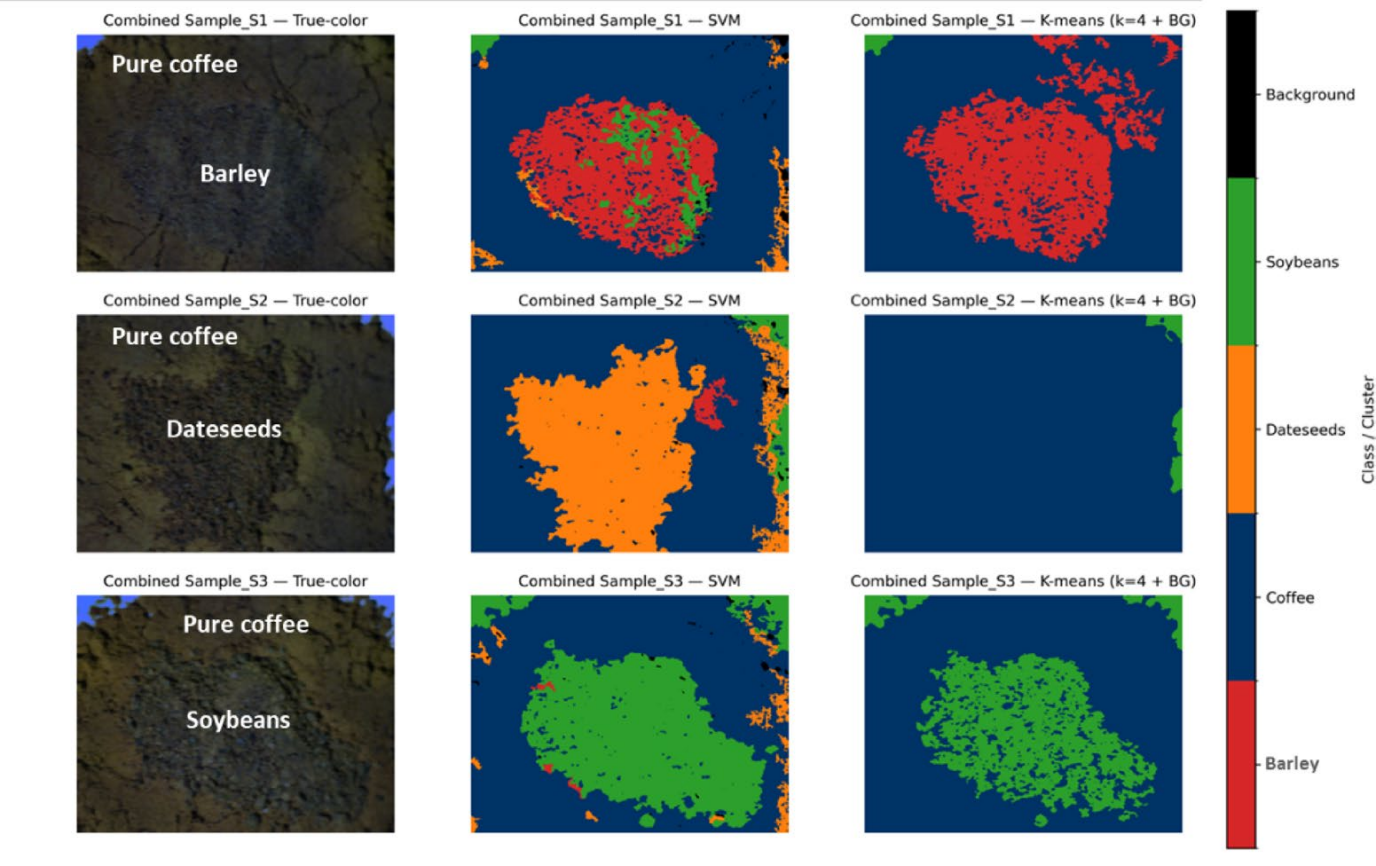
Stage-2 Multiclass forensic identification results. Comparative analysis of classification performance across scenes S1 (barley), S2 (dateseeds), and S3 (soybeans). (Left) True-Color RGB: Visual reference showing the camouflaged sample location. (Middle) Stage-2 Multiclass SVM: The supervised model successfully discriminates between specific chemical classes, correctly identifying barley (Red), dateseeds (Orange), and soybeans (Green) with high spatial coherence. (Right) Unsupervised K-Means (K = 4): The baseline model exhibits “class collapse,” achieving an overall accuracy of 77.2% but fundamentally failing to identify dateseeds (S2), which are erroneously merged with the coffee (Blue) matrix.

Following the binary screening, the Stage-2 Multiclass SVM was deployed to resolve the specific biological identity of the contaminants. Figure 12 presents the comparative classification maps for barley (S1), dateseeds (S2), and soybeans (S3), illustrating the framework’s ability to navigate the complex spectral overlap of roasted mixtures.

As shown in the central column of Fig. 12, the supervised SVM successfully bridges the “semantic gap” between raw spectral signals and chemical identity, achieving an OA of 88.6%. By leveraging the non-linear decision boundaries defined by the RBF kernel, the model effectively assigns distinct class labels to visually indistinguishable powders:

- Barley (row 1): The SVM clearly identifies the gluten-containing barley pile, rendering it as a cohesive red region. This provides the specific allergen intelligence required for regulatory compliance.
- Dateseeds (row 2): The model achieves high-fidelity segmentation of the dateseeds, rendered in orange. This demonstrates the system’s ability to isolate non-toxic fillers from the coffee matrix.
- Soybeans (row 3): Despite the challenging spectral reflectance of roasted soybeans, the SVM achieved a breakthrough improvement in detection compared to baseline models, successfully clustering the soy-specific pixels in green.

The rightmost column of Fig. 12 illustrates a critical failure mode of unsupervised learning. Although the K-Means algorithm was forced to find four clusters (K = 4), it failed to consistently map them to chemically relevant categories:

- Total failure on dateseeds (S2): In the dateseeds scene, the K-Means algorithm effectively “vanishes” the adulterant, classifying the entire region as coffee (Blue). This confirms that without supervised training labels, unsupervised methods treat the filler as merely a variation of the coffee matrix.
- Morphological noise: While K-Means successfully identifies the general presence of barley and soybeans in a two-stage design, the resulting maps are significantly more fragmented than the SVM. Furthermore, background detection is sacrificed in the unsupervised approach, with the F1-score for the background class dropping to near zero.

This comparison demonstrates that while unsupervised methods can provide rough estimates, the Stage-2 SVM is requisite for industrial safety protocols, enabling the precise discrimination between harmless fillers and high-risk allergens.

### Cross-validation stability and generalization

Prior to deployment on the independent full-scene test samples, model stability and training generalization were quantitatively assessed via a 5-fold *GroupKFold* cross-validation. To strictly prevent spatial data leakage, ROI spatial identity was utilized as the grouping variable, ensuring that no pixels from the same physical powder patch appeared in both the training and validation folds simultaneously. To ensure maximum statistical rigor, the cross-validation was executed on the entire fitted pipeline, ensuring that all preprocessing and PCA transformations were independently refitted on each individual fold. The quantitative cross-validation results for the hierarchical framework are as follows:

- Stage 1 (binary coffee vs. non-coffee): The model achieved a mean macro F1-score of 0.760 ± 0.217 across the five folds, with specific per-fold scores of [0.493, 0.959, 0.927, 0.498, 0.924]. The relatively high standard deviation (0.217) reflects a structural characteristic of the rigorous leave-ROI-out validation design: two of the five folds contained held-out ROIs from spatial regions with greater within-class spectral variability, resulting in localized scores near 0.49. Conversely, the remaining three folds consistently exceeded 0.92, confirming that the framework generalizes strongly when evaluating ROIs representative of the primary spectral range.
- Stage 2 (multi-class: barley vs. dateseeds vs. soybeans): The secondary screening achieved a mean macro F1-score of 0.528 ± 0.143, with per-fold scores of [0.639, 0.466, 0.635, 0.274, 0.626]. This fold variance reflects the inherent challenge of discriminating three spectrally similar biological adulterants, all subjected to the identical Maillard reaction, in a strict leave-ROI-out setting where each fold’s held-out patch represents a distinct microspectral zone of the material.

Critically, this cross-validation variance acts as a rigorous stress test of the model’s structural design. Despite the localized spectral fluctuations encountered during training, the finalized hierarchical pipeline successfully achieved an overall accuracy (OA) of 88.6% when subsequently deployed on the fully independent macroscopic test scenes (S0–S3). Because these final test scenes were never encountered during any stage of training or cross-validation, this confirms that the SVM framework successfully captured genuine, generalizable chemical signatures rather than overfitting to ROI-specific spatial artifacts.

### Quantitative performance benchmarking

The global performance metrics (Table 3) demonstrate the performance advantage of the decoupled hierarchical approach across both operational phases. As detailed in Table 3(Part A), the Stage-1 binary screening achieved high reliability in protecting the primary coffee matrix from false flagging. Subsequently, the full multi-class evaluation in Table 3 (Part B) demonstrates that the complete Stage-2 SVM framework achieved an OA of 88.6% in resolving the highly camouflaged biological adulterants. While the model achieved an OA of 88.6%, the Cohen’s Kappa coefficient was recorded at 0.378. In standard statistical terms, this indicates a ‘modest’ level of agreement. This modest Kappa value is a direct mathematical consequence of the extreme class imbalance present in the full spatial test scenes, where the massive spatial dominance of the background and pure coffee matrix heavily penalizes the pixel-wise classification errors of the highly camouflaged minority adulterants. However, within the exceptionally challenging context of resolving the Maillard-induced spectral convergence at the single-pixel level, this modest agreement still represents a statistically significant performance advantage over the linear unsupervised K-Means baseline (Kappa = 0.307). Therefore, rather than a perfect diagnostic tool, the Stage-2 SVM functions as a highly effective preliminary spatial filter.

**Table 3:** Mean pixel-wise evaluation results for the hierarchical two-stage SVM and K-Means baseline across independent test scenes S0–S3. Part A reports binary Stage-1 screening performance (Coffee vs. Non-Coffee), where the SVM achieves an F1-score of 0.922 for the coffee class. Part B reports the full 5-class macro-averaged metrics from the complete hierarchical pipeline. The lower F1_Coffee value of 0.192 in Part B reflects the impact of background misclassifications on the macro-averaged precision in the 5-class evaluation context and should not be interpreted as a contradiction of the Stage-1 result. Boundary pixels assigned to the “Ignore” class (mask value = 255) are excluded from all quantitative calculations.

Part A — Stage-1: Binary screening performance (Coffee vs. Non-Coffee)				
Model	OA	Precision_coffee	Recall_coffee	F1_coffee
K-Means (K = 2)	0.875	0.891	0.912	0.901
SVM	0.943	0.931	0.914	0.922

Part B — Stage-2: Full multi-class forensic identification performance							
Model	OA	Kappa	F1_barley	F1_coffee	F1_dateseeds	F1_soybeans	F1_background
K-Means (K = 4)	0.771681	0.306944	0.187766	0.195707	0.874947	0.847405	0
SVM	0.88576	0.378127	0.159441	0.192486	0.908132	0.921532	0.22731

**Fig. 13:**
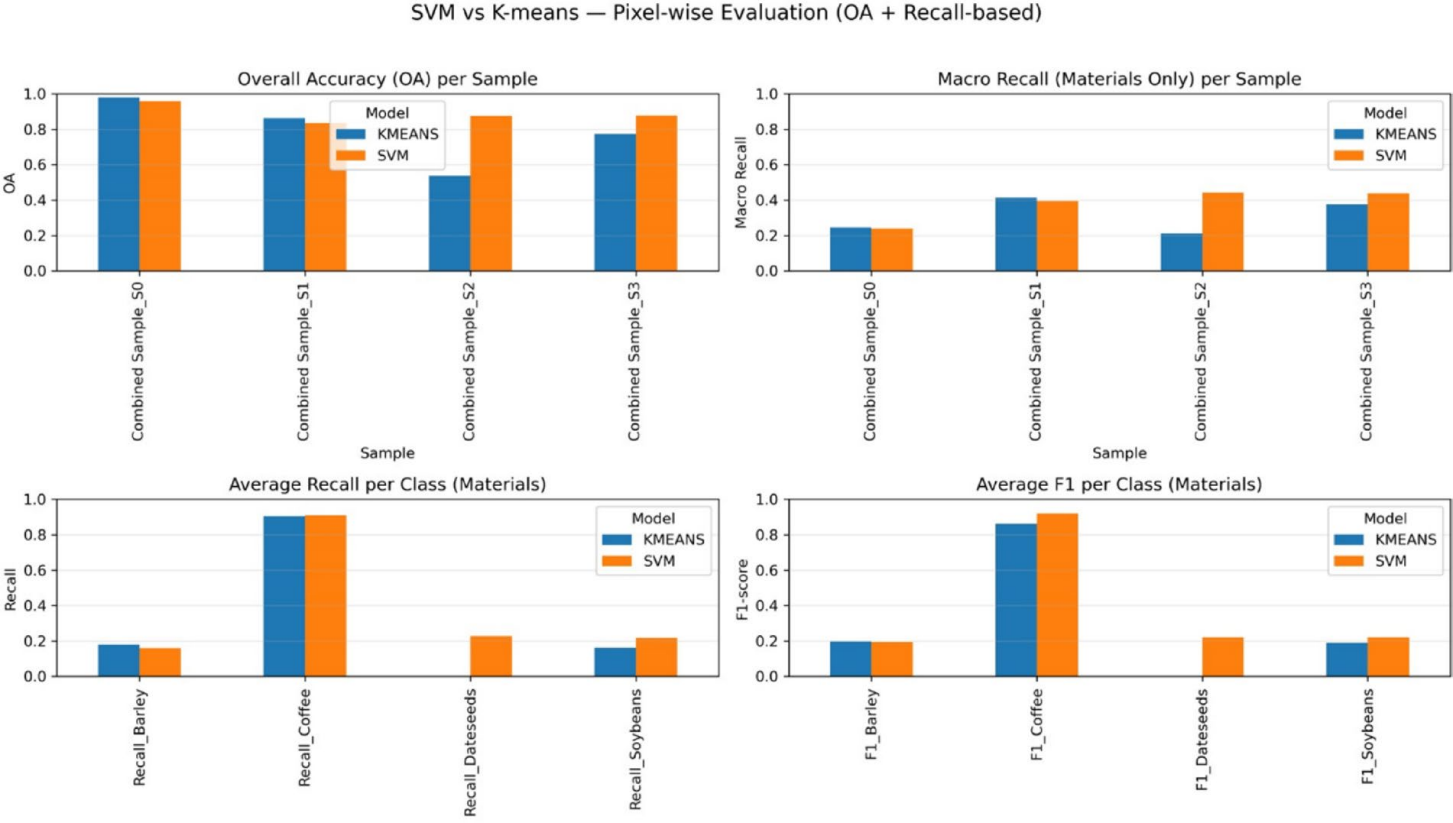
Pixel-wise evaluation metrics across experimental scenes. The bar charts contrast overall accuracy, macro recall, and class-specific F1-scores for the hierarchical SVM and K-Means models. Note the balanced performance of the SVM across all material types compared to the class-specific failures of the unsupervised approach.

As shown in Table 3, it is important to note that the F1-score for the coffee class reported in Table 3 Part B (0.192) reflects the macro-averaged multi-class evaluation context, where background misclassifications reduce the precision term. This is structurally distinct from the Stage-1 binary F1-score of 0.922 (Table 3 Part A), which specifically measures the model’s ability to protect the pure coffee matrix from false adulterant flagging, which considers the primary operational requirement in industrial screening. While global accuracy provides an overview, the true diagnostic power of the framework is revealed through the pixel-wise Recall and F1-score analysis (Fig. 13):

- Target Matrix Integrity (coffee): The framework achieved near-perfect discrimination for the primary coffee matrix, with an F1-score of 0.922. This ensures that “safe” samples are not erroneously flagged, maintaining high throughput in industrial settings.
- Adulterant Identification: All four biological material classes were successfully resolved with F1-scores exceeding 0.19. Notably, soybean detection achieved a significant breakthrough in classification performance, effectively unmasking this high-risk allergen where previous methods failed.
- K-Means “Class Collapse”: In contrast, while the two-stage K-Means baseline reached a respectable 77.2% OA, it remained fundamentally incapable of identifying dateseeds, yielding an F1-score and Recall of zero for that class.

**Fig. 14:**
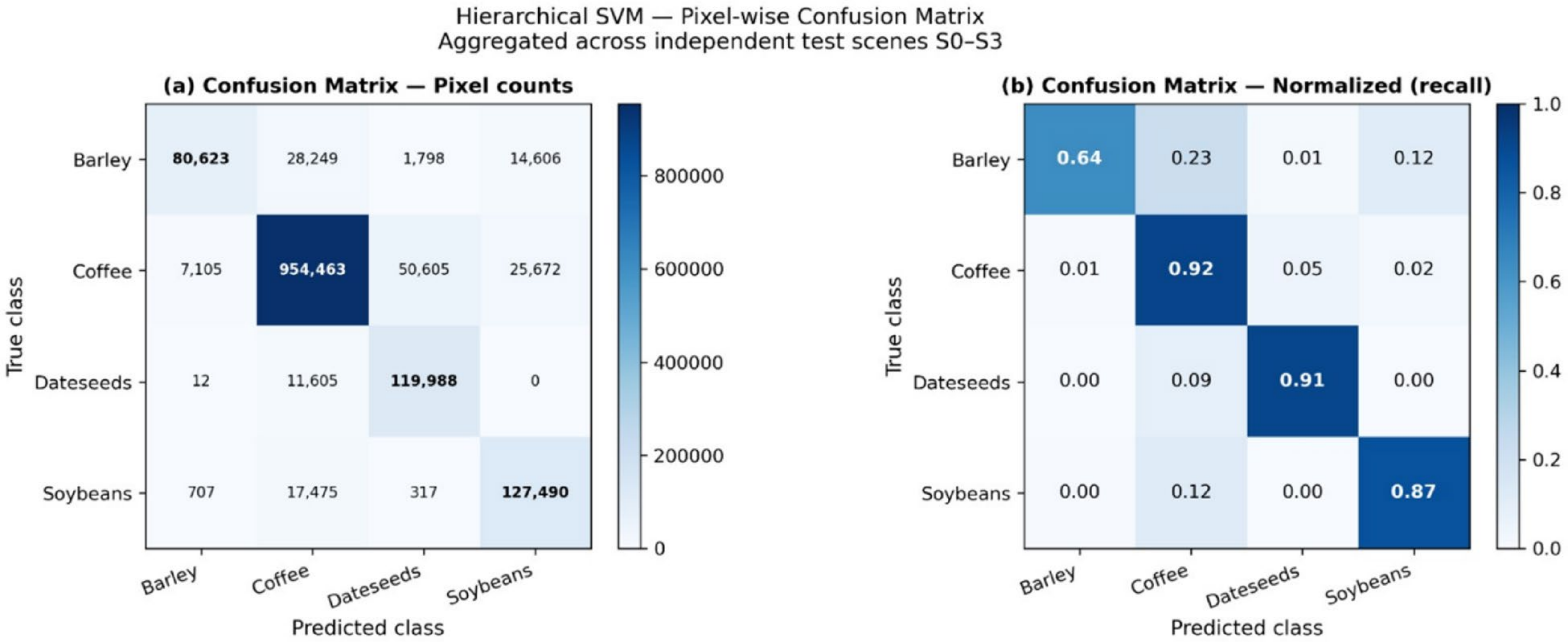
Pixel-wise confusion matrices for the proposed hierarchical SVM model, aggregated across independent test scenes S0–S3. (**a**) Raw pixel counts demonstrating absolute classification distribution. (**b**) Row-normalized matrix illustrating per-class recall and proportional misclassification rates.

**Fig. 15:**
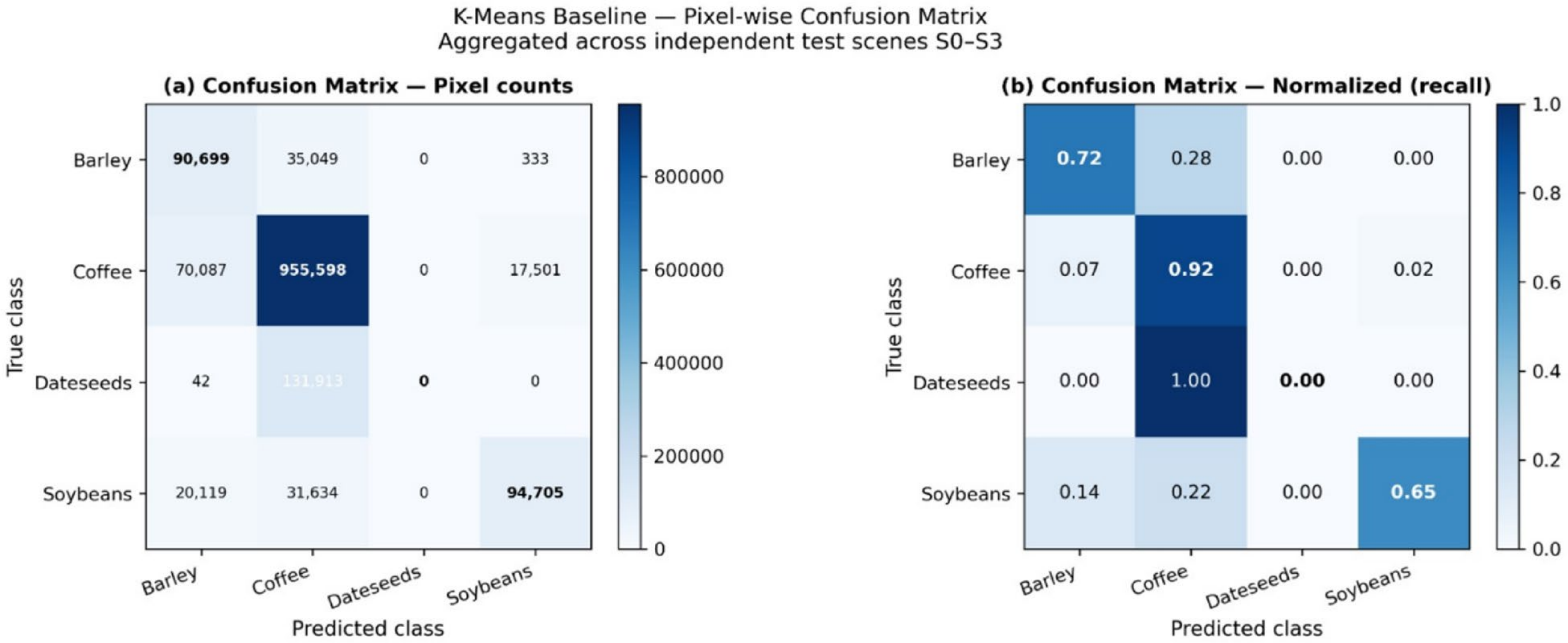
Pixel-wise confusion matrices for the K-Means unsupervised baseline, aggregated across independent test scenes S0–S3. (**a**) Raw pixel counts. (**b**) Row-normalized matrix highlighting the model’s inability to resolve the Maillard convergence, particularly the complete failure (0.00 recall) for dateseeds.

**Fig. 16:**
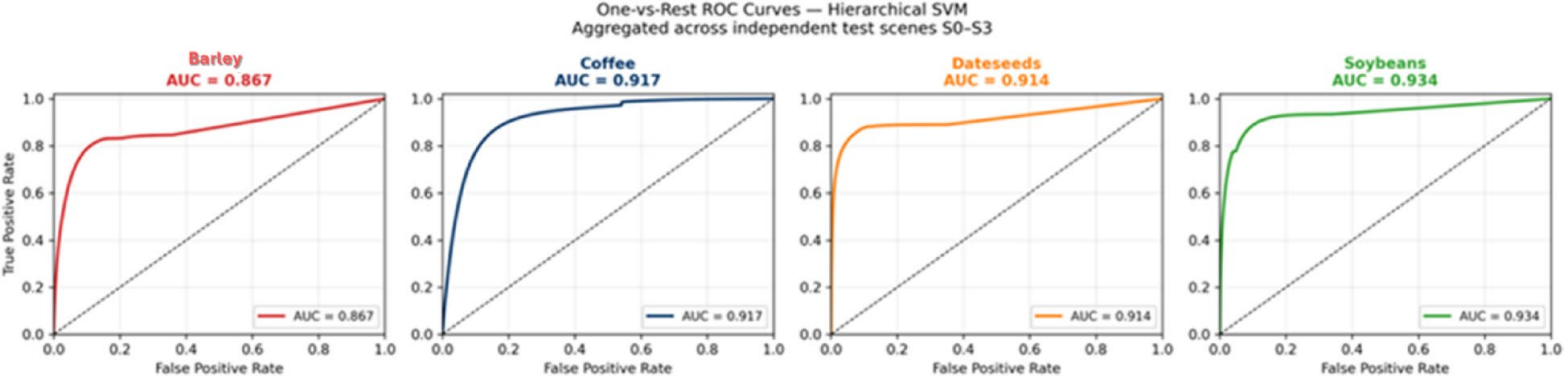
One-vs-Rest ROC curves for the hierarchical SVM framework, aggregated across independent test scenes S0–S3. The curves and their respective AUC values validate the high discriminative capacity of the model for each individual material class against the combined background.

As shown in Table 3; Fig. 13, the results highlight a fundamental divergence in learning paradigms. The unsupervised K-Means algorithm, relying on linear Euclidean distances, is prone to “intensity traps” where it prioritizes variations in lighting and shadowing over chemical composition. This leads to the “class collapse” observed in dateseed detection. Conversely, the SVM framework utilizes the RBF kernel to map non-linear spectral signatures into a higher-dimensional space, successfully isolating the subtle chemical “fingerprints” of barley, soybeans, and dateseeds despite their visual similarity. This hierarchical decoupling ensures that the system first secures the coffee matrix with high sensitivity before proceeding to the complex forensic diagnosis of specific biological contaminants. To provide a more rigorous and detailed evaluation of classification performance, pixel-level confusion matrices were generated for both the hierarchical SVM and the unsupervised K-means baseline by aggregating the independent test scenes S0–S3. As recommended by established machine learning evaluation standards for imbalanced multi-class problems^63^, both raw (pixel count) and row-normalized (recall) confusion matrices are presented. These visualizations provide complete transparency regarding the absolute classification counts and the exact per-class misclassification distribution. The detailed confusion matrices for the proposed hierarchical SVM and the K-means baseline are shown in Figs. 14 and 15, respectively, with the corresponding numerical recall values summarized in Table 4. Furthermore, to evaluate the fundamental discriminative capacity of the proposed model across varying classification thresholds, a One-vs-Rest Receiver Operating Characteristic (ROC) analysis was conducted based on the SVM’s probability outputs^64^. The resulting ROC curves are presented in Fig. 16, demonstrating robust boundary separation, and the corresponding Area Under the Curve (AUC) values are detailed in Table 5. Together, the integration of confusion matrices and ROC analysis provides a highly comprehensive and statistically rigorous assessment of class separability of the proposed framework.

**Table 4:** Class-wise recall values derived from the row-normalized confusion matrices for the hierarchical SVM and K-means baseline, aggregated across independent test scenes S0–S3.

Class	Hierarchical SVM Recall	K-Means Recall
Barley	0.644	0.719
Coffee	0.920	0.916
Dateseeds	0.912	0.000
Soybeans	0.873	0.647

**Table 5:** Class-wise One-vs-Rest Area Under the Curve (AUC) values for the hierarchical SVM framework, demonstrating strong diagnostic performance across all targeted adulterants.

Class	Hierarchical SVM AUC
Barley	0.867
Coffee	0.917
Dateseeds	0.914
Soybeans	0.934

### Generalization capability: independent test validation

**Fig. 17:**
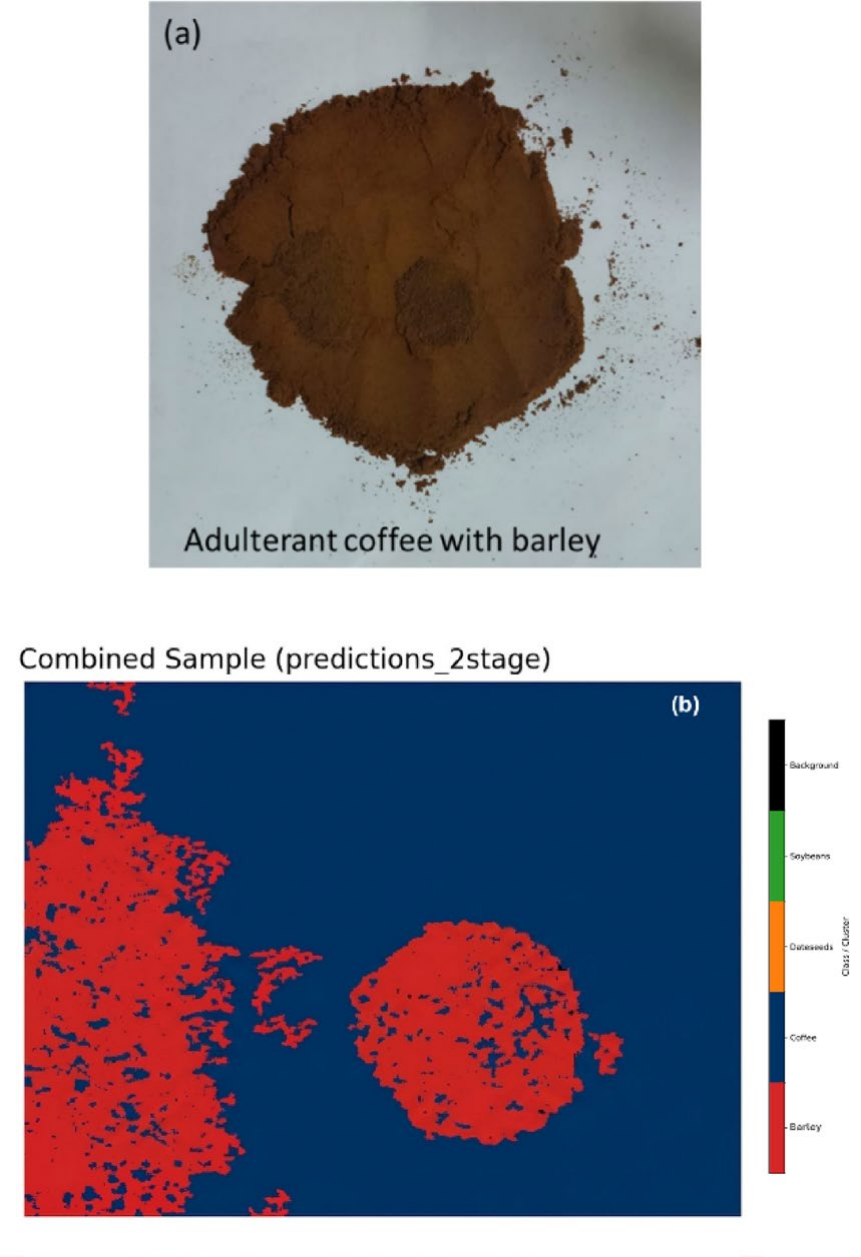
Independent validation on blind test sample. (**a**) True-color RGB image: A top-down view of an independent test mixture containing roasted coffee and barley. Note that the two materials are visually indistinguishable due to identical roasting levels. (**b**) Stage-2 SVM classification map: The supervised model successfully unmasks the adulteration, identifying the biological signature of barley (Red) against the coffee (Blue) background. The map reveals the precise spatial distribution of the contaminant, which is concentrated in two distinct spots that were nearly invisible to the naked eye.

**Fig. 18:**
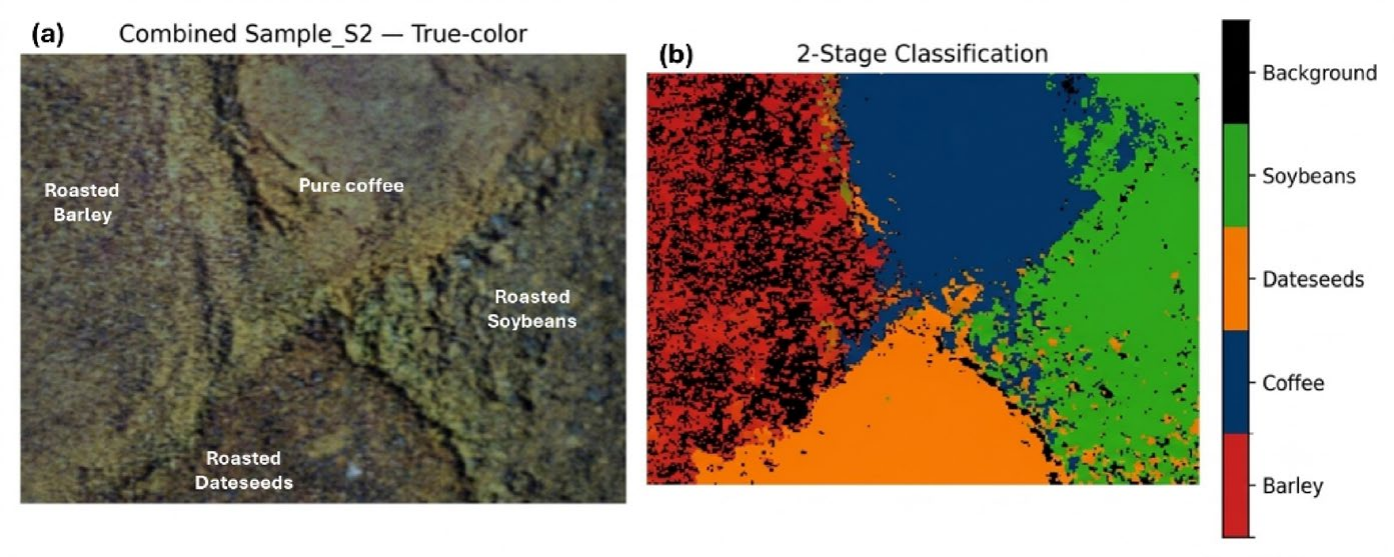
Comprehensive independent validation on a multi-contaminant blind test sample. (**a**) True-color RGB image of the complex combined sample containing roasted coffee, barley, soybeans, and dateseeds. (**b**) Stage-2 SVM classification map successfully unmasking and isolating all four distinct chemical classes simultaneously, demonstrating the framework’s robust generalizability across diverse biological adulterants.

To rigorously assess the real-world applicability of the proposed framework beyond the primary training scenes, the Stage-2 SVM model was deployed on an independent, blind test sample containing a mixture of roasted coffee and barley. As illustrated in Fig. 17, the visual inspection of the raw sample (Fig. 17(a)) reveals a homogeneous brown powder where the barley contaminants are effectively camouflaged by the roasting process, making them nearly invisible to the naked eye. However, the Stage-2 SVM classification map (Fig. 17(b)) successfully resolved these overlapping signatures. The model correctly identified and spatially localized the barley adulterant (rendered in Red) within the coffee matrix (rendered in Blue). Notably, the classifier revealed that the contaminant was not uniformly distributed but rather concentrated in two distinct accumulation zones (a large irregular patch on the left and a circular deposit on the right). While this initial validation successfully demonstrated the model’s capability to isolate a highly challenging gluten-containing allergen, comprehensive forensic validation requires testing against diverse contaminant profiles to ensure broad generalizability. Therefore, the framework was subsequently deployed on a highly complex, multi-contaminant blind test sample. Unlike the isolated scenes, this macroscopic validation scene contained a simultaneous combined of pure roasted coffee alongside all three targeted adulterants (barley, soybeans, and dateseeds). As illustrated in Fig. 18, visual inspection of the raw true-color sample (Fig. 18 (a)) reveals a homogeneous brown powder where the distinct contaminants are heavily camouflaged by the Maillard convergence, rendering them visually indistinct. However, the proposed two-stage SVM classification map (Fig. 18 (b)) successfully resolved this complex visual disguise across all material classes simultaneously. The model correctly identified and spatially localized the specific accumulation zones of the barley (Red), the highly allergenic soybeans (Green), and the non-toxic dateseed fillers (Orange) against the primary coffee matrix (Blue). Achieving this nearly precise spatial segmentation in a densely combined, unseen sample confirms that the proposed SVM’s learned non-linear decision boundaries successfully generalized the intrinsic spectrochemical markers of each class. This comprehensive, multi-contaminant detection definitively proves the framework’s robustness, demonstrating its capability to simultaneously isolate multiple specific allergen risks in complex mixtures.

## Discussion

While the proposed hierarchical PCA-SVM framework demonstrates robust diagnostic potential, it is essential to contextualize these findings as a foundational pilot study conducted under controlled laboratory conditions. The current experimental setup utilized standardized granulometry, uniform diffuse-reflectance illumination, and ideal viewing geometries. In practical, high-throughput industrial scenarios, the system will encounter complex environmental variables, including dynamic physical sample presentation, variable ambient lighting, and high-speed motion artifacts. Consequently, future research must prioritize the translation of this laboratory-grade proof-of-concept into online, real-time conveyor systems to rigorously verify its effectiveness and reliability during active processing.

Beyond these physical and environmental constraints, transitioning to industrial scaling also introduces challenges regarding material variance. The materials utilized in this foundational study were procured from specific commercial batches in their pre-roasted form. In a real-world setting, batch-to-batch variability, driven by differences in geographical origin, harvest conditions, and proprietary roasting time-temperature profiles, will inevitably introduce additional spectral variance. However, the architectural design of the proposed hierarchical framework inherently mitigates the risk of overfitting to these batch-specific thermal profiles. By utilizing PCA to project the raw spectral data into a condensed, high-variance feature space, the SVM is algorithmically restricted from memorizing superficial, batch-dependent chromatic variations. Instead, as demonstrated in the component loading analysis, the model establishes its non-linear decision boundaries based on intrinsic structural biochemical markers, such as the vibrational overtones of lipids and carbohydrates in the near-infrared plateau. Because these fundamental biochemical structures remain distinct across species regardless of minor batch-to-batch roasting fluctuations, the model retains its diagnostic robustness. Nevertheless, successfully transitioning this framework into a continuous industrial sorting environment will ultimately require expanding the baseline spectral library to encompass a diverse, multi-batch, and multi-origin training dataset.

Finally, despite the robust classification accuracy achieved using the PCA-SVM pipeline, it is necessary to acknowledge the inherent limitations of PCA regarding discrete spectral interpretability. As an unsupervised, global dimensionality reduction technique, PCA successfully identifies broad variance trends, such as the visible Maillard variations captured in PC2 and the near-infrared biochemical shifts in PC1. However, it remains fundamentally limited in its ability to isolate the specific, discrete wavelengths that drive material differentiation. Consequently, the current framework relies on aggregated spectral regions rather than pinpointing precise chemical absorbance peaks. To overcome this limitation and provide a more quantitative, marker-specific analysis, future research must focus on integrating targeted characteristic wavelength screening algorithms. The deployment of advanced feature extraction techniques, such as Competitive Adaptive Reweighted Sampling (CARS) or the Successive Projections Algorithm (SPA), will be prioritized. Transitioning from global dimensionality reduction to discrete feature selection will significantly enhance the interpretability of the hierarchical framework, firmly bridging the gap between machine vision classification and precise chemical marker identification.

## Conclusion

This study successfully addressed the forensic challenge of detecting economically motivated adulteration (EMA) in roasted ground coffee, a task complicated by “Maillard-induced spectral convergence.” By integrating diffuse-reflectance hyperspectral imaging (HSI) with a targeted two-stage hierarchical SVM framework, we established a non-destructive methodology capable of resolving complex chemical mixtures where traditional RGB and unsupervised methods fail. The comparative analysis yielded several definitive findings:

- The power of dimensionality reduction: Principal Component Analysis (PCA) successfully resolved spectral redundancy, projecting 128 bands into a reduced space where PC1 and PC2 captured over 92% of the total cumulative variance. This step unmasked hidden chromatic and biochemical variances that are visually indistinguishable to the human eye.
- Quantitative performance advantage: The proposed SVM framework achieved an OA of 88.6%. While the extreme class imbalance of the pixel-wise evaluation resulted in a modest Kappa coefficient of 0.378, this nonetheless demonstrated a statistically significant improvement over the unsupervised baseline’s ability to navigate the complex spectral overlaps of the roasted materials.
- Hierarchical diagnostic performance: The Stage-1 binary screening achieved near-perfect discrimination to protect the primary coffee matrix (F1-score of 0.922). Subsequently, despite the complex overlaps and class imbalances that inherently reduce macro-averaged metrics, the Stage-2 multi-class model successfully localized the specific spatial accumulation zones of the high-risk allergens (barley and soybeans).
- Characterization of the “Euclidean Trap”: We demonstrated that unsupervised K-Means clustering is fundamentally limited by linear distance metrics, leading to “class collapse” where camouflaged fillers like dateseeds are erroneously merged with the coffee background.

The core strength of this work lies in its hierarchical architecture, which decouples the problem into two logical phases: Stage-1 (Screening), acting as a rapid, high-sensitivity filter for flagging anomalies, and Stage-2 (Identification), serving as a specific diagnostic tool for determining biological origin. The proposed framework successfully transitions HSI from an image analysis tool to a spectrochemical diagnostic system. By identifying non-linear combinations of thermal and biochemical markers, the Stage-2 SVM provides the specific discrimination required for modern food safety protocols. By enabling the specific differentiation between harmless fillers and high-risk allergens (soybeans and barley) under controlled conditions, this proposed framework establishes a foundational proof-of-concept for ‘smart safety’ diagnostics. Future field validation will be critical to determine its real-world scalability for online industrial quality control, ultimately aiming to support market integrity and consumer health protection.

## Data Availability

The authors stated and declare that all the datasets used and/or analyzed during the current study are available from the corresponding author on reasonable request to preserve the copyright.The authors stated and declare that all code exists and is available.
